# Workplace mental health screening: a systematic review and meta-analysis

**DOI:** 10.1136/oemed-2022-108608

**Published:** 2023-06-15

**Authors:** Jessica Strudwick, Aimee Gayed, Mark Deady, Sam Haffar, Sophia Mobbs, Aiysha Malik, Aemal Akhtar, Taylor Braund, Richard A Bryant, Samuel B Harvey

**Affiliations:** 1 Black Dog Institute, University of New South Wales, Randwick, New South Wales, Australia; 2 Faculty of Medicine, University of New South Wales, Kensington, New South Wales, Australia; 3 Department of Mental Health and Substance Use, World Health Organization, Geneve, Switzerland; 4 School of Psychology, University of New South Wales, Sydney, New South Wales, Australia; 5 Division of Insurance Medicine, Department of Clinical Neuroscience, Karolinska Institute, Stockholm, Sweden

**Keywords:** Occupational Health, Mental Health, Public Health Surveillance

## Abstract

Workplaces are an important location for population mental health interventions. Screening to detect employees at risk of or experiencing mental ill health is increasingly common. This systematic review and meta-analysis examined the efficacy of workplace mental health screening programmes on employee mental health, work outcomes, user satisfaction, positive mental health, quality of life, help-seeking and adverse effects. PubMed, PsycINFO, EMBASE, CENTRAL, Global Index Medicus, Global Health and SciELO were searched (database inception–10 November 2022) and results screened by two independent reviewers. Controlled trials evaluating screening of workers’ mental health as related to their employment were included. Random effects meta-analysis was performed to calculate pooled effect sizes for each outcome of interest. Grading of Recommendations Assessment, Development and Evaluation was conducted to evaluate the certainty of findings. Of the 12 328 records screened, 11 were included. These reported 8 independent trials collectively assessing 2940 employees. Results indicated screening followed by advice or referral was ineffective in improving employee mental health symptoms (n=3; d=−0.07 (95% CI −0.29 to 0.15)). Screening followed by facilitated access to treatment interventions demonstrated a small improvement in mental health (n=4; d=−0.22 (95% CI −0.42 to –0.02)). Limited effects were observed for other outcomes. Certainty ranged from low to very low. The evidence supporting workplace mental health screening programmes is limited and available data suggest mental health screening alone does not improve worker mental health. Substantial variation in the implementation of screening was observed. Further research disentangling the independent effect of screening alongside the efficacy of other interventions to prevent mental ill health at work is required.

**WHAT IS ALREADY KNOWN ON THIS TOPIC**

- Mental health interventions are increasingly implemented in workplaces to protect and promote employee mental health. Screening for the early detection of employee mental health conditions is one theoretically acceptable approach. Despite the increasing use of this approach in workplaces, the efficacy of this approach to improve employee mental health is unclear.

**WHAT THIS STUDY ADDS**

- This review identified limited research evaluating workplace mental health screening programmes. Available data suggest that screening followed by feedback and advice does not improve employee mental health. However, screening followed by allocation to specific treatment interventions had a more promising impact on employee mental health.

**HOW THIS STUDY MIGHT AFFECT RESEARCH, PRACTICE OR POLICY**

- Given the limited research available, screening should not be used as a primary or stand-alone approach to mental health in the workplace. Practitioners and policy-makers should be cognisant of the lack of evidence supporting this type of intervention when encouraging the use of workplace mental health interventions to protect employee mental health. Future research that examines screening separate from subsequent interventions is required before workplace mental health screening can be recommended as an early intervention for employee mental health. Confidentiality is a key consideration for both organisations administering screening and researchers conducting screening experiments. Exactly who has access to employee reported mental health data should be clearly communicated to employees.

## Introduction

Depressive and anxiety disorders are among the leading causes of overall disease burden in working aged individuals globally.^1^ Concurrently, mental health conditions are increasingly recognised as a leading cause of sickness absence and incapacity benefits in high-income countries.^2–7^ In Great Britain during 2019/2020, work-related depression, anxiety or stress accounted for over half of all cases of work-related ill health.^7^ Depressive symptoms are strongly associated with presenteeism and poor job performance.^8–12^ Therefore, mental ill health experienced by working individuals represents a considerable burden to individuals, workplaces and society at large.^13^ As a result, workplaces are increasingly recognised as a critical platform to implement population mental health interventions.^14 15^ While policy-makers globally are encouraging and/or mandating workplaces to implement mentally healthy practices, there remains uncertainty surrounding what workplace interventions are likely to improve mental health outcomes.

Many mental health interventions implemented within the workplace are reactive, targeting symptomatic employees already experiencing sickness absence. However, there is a growing interest in the possibility that workplace interventions may be able to aid early detection of mental health conditions^16^ facilitating timely prevention. One common early intervention approach is mental health screening. Screening is the process of assessing a population of individuals to identify unrecognised early disease, precursors of disease or the disease itself.^17^ Workplace mental health screening involves assessing the mental health of employees and detecting those at risk of or experiencing a mental health condition, and in turn, encouraging help-seeking, treatment or some other form of appropriate response.

Many individuals experiencing mental ill health may not recognise their symptoms as mental illness resulting in delayed access to treatment, often seeking help only after symptom severity escalates to the point of major functional impact.^18 19^ Early detection of mental ill health is also associated with less intensive interventions and improved recovery.^20–22^ When used in the workplace, screening may prevent employees’ mental health deteriorating to the point where they experience sickness absence, in turn reducing incapacity of a workforce. While there are logical reasons that mental health screening could be an effective intervention, it is not without criticism due to varying degrees of uptake and rates of false positives.^17 23^ Indeed, a recent practice review modelled a 14% false positive rate for depression screening in primary care.^24^ The review also identified four recent trials evaluating depression screening, reporting mixed results that suggested no effect on mental health symptoms. Furthermore, screening has the potential for harm due to risks associated with labelling transient distress as illness, stigma associated with depression, and making completers feel more unwell and overfocused on their symptoms. In the workplace, these complexities are further complicated by employee concerns regarding confidentiality and perceived consequences of disclosing their mental health status.^25 26^

Given these issues, determining whether workplace mental health screening is effective and elicits benefits that outweigh respective risks, is critical. A large meta-review of workplace mental health interventions highlighted that the evidence base for workplace mental health screening has never been directly addressed in any moderate/high-quality reviews.^14^ Another review focusing on high-risk occupational groups (ie, military, police and rescue workers) identified only three studies describing work health surveillance (WHS) in relation to psychological screening, despite identifying 22 studies assessing physical or environmental exposures.^27^ Further, a very large randomised controlled trial (RCT) of UK military personnel failed to detect any beneficial effect of postdeployment mental health screening on mental health symptoms and help-seeking behaviours 10–24 months later.^28^

Despite these uncertainties, workplace mental health screening is increasingly implemented in real-world workplaces across many different industry groups without an evidence base for its effectiveness. Clarity about what, if any, impact mental health screening in the workplace has on employee outcomes is urgently required to ensure implementation of this population mental health intervention has value in the workplace. The present review aims to identify and synthesise the existing evidence describing the efficacy of workplace mental health screening in reducing mental health symptoms in workers. It also seeks to examine the effect of screening on work-related outcomes, users’ satisfaction with screening, positive mental health, quality of life, help-seeking and the occurrence of any adverse effects.

## Methods

This review protocol was registered on the PROSPERO International Prospective Register of Systematic Reviews (CRD42021248725). This review forms one of the evidence reviews which were commissioned for a WHO Guideline Development Group to develop global guidelines on mental health at work. The review was conducted in line with the Preferred Reporting Items for Systematic Reviews and Meta-Analyses 2020 statement.^29^

### Study selection criteria

Included studies were limited to controlled trials of employees (aged ≥18 years) undergoing mental health screening conducted at or by a workplace or as related to their employment. All trials had to specify screening as the primary component of the study and all types of screening identified by the search were considered if they included an assessment of mental health. Trials where participants were allocated to subsequent interventions based on the results of screening were also eligible for inclusion. However, trials that involved screening as one component (ie, a baseline questionnaire) with a primary focus on the evaluation of another specific mental health intervention (ie, mindfulness programme, exercise) where participants were allocated to treatment or control group(s) irrespective of their screening/baseline questionnaire results were ineligible. The rationale for this criterion was that this review aimed to disentangle effects of mental health screening specifically, and given it is commonplace in workplace intervention research to refer to baseline assessments as ‘screening’, it was critical to include only those trials where screening was the driver of any detectable intervention effects. Employees receiving screening interventions had to be compared with business/care as usual, another intervention or no intervention. Further, trials had to measure and report at least one of the following outcomes of interest: mental health symptoms, work-related outcomes (such as sickness absence, work functioning, performance), user satisfaction, positive mental health, quality of life, help-seeking or adverse effects (including participant drop-out and participant reported incidents of harm).

Trials evaluating screening for sleep disorders, neurological conditions (ie, dementia, epilepsy, head injury, cognitive and intellectual disabilities) or substance use (ie, alcohol, drug) were excluded. Trials assessing pre-employment screening and those involving military, forced labour, child labour, trafficking and modern slavery, illicit work, volunteer or unpaid worker populations were also excluded. No exclusion criteria for year of publication or language were imposed. Registered trials not published in peer-reviewed journals were eligible for inclusion, and if meeting inclusion criteria, trial representatives were contacted to provide data. If no response was received after 2 weeks, or if outcome data were not available, the trial was excluded.

### Search strategy

To identify trials, PubMed, PsycINFO (Ovid), EMBASE (Ovid), Cochrane Central Register of Controlled Trials (CENTRAL), Global Index Medicus, Global Health and SciELO were searched from inception to 11 March 2021 using the search terms provided in online supplemental information 1. No restrictions or filters were placed on searches. On 11 May 2021, the reference lists of included trials were handsearched, forward citation searching using Google Scholar was conducted, and the first 20 pages of Google Scholar were searched using ‘workplace mental health screening’ to detect any additional studies. Database searching was updated on 10 November 2022. Studies identified via 2021 searching informed the WHO Guideline Development Group to develop global guidelines on mental health at work, while this manuscript is informed by all studies identified up to 10 November 2022.

10.1136/oemed-2022-108608.supp1 Supplementary data

### Screening and data extraction

Endnote V.X9 and Covidence systematic review software were used for screening and extraction procedures. Search results from each database were uploaded into Endnote V.X9 and duplicates removed and checked. Then, two researchers who received training and supervision before and throughout the systematic review procedure independently screened all titles and abstracts for eligibility. Records that appeared relevant were uploaded into Covidence for full-text screening, individually by the same two researchers, for final inclusion in the review. A third researcher (JS, AG and SBH) was consulted regarding records for which consensus was not reached.

Once the final set of included articles was decided, descriptive data extraction was independently conducted by the two researchers who conducted screening procedures. The following characteristics from each included study were extracted: (1) author and year published, country the study was conducted in, start and end date, (2) population including occupation/sector of employment, employment type (ie, informal/formal, part time/full time), demographics, sample size, proportion with a mental health issue at baseline, (3) study design, response rate, inclusion/exclusion criteria, (4) intervention information including length, description of intervention and control, who delivered the intervention, and mode of delivery, (5) outcome variables including measures used, follow-up length, lost to follow-up and (6) findings related to the outcomes of interest. Extracted data were compared and discrepancies were resolved by a third researcher (JS, AG and SBH).

### Risk of bias and evidence certainty assessment

Two researchers independently assessed the risk of bias associated with the primary outcome for each included study. Any discrepancies were resolved by discussion with a third researcher (JS, AG and SBH). The Cochrane risk-of-bias tool for randomised trials (RoB 2.0) and the additional guidance for cluster randomised trials^30^ was used. RoB 2.0 rates bias as either low, some concerns or high due to randomisation, deviations from intended intervention, missing outcome data, outcome measurement and the selection of the reported result. Certainty of the evidence supporting each finding was assessed by three authors (JS, RAB and SBH) using the Grading of Recommendations Assessment, Development and Evaluation approach.^31^ Certainty scores began at ‘high’ and were downgraded incrementally to ‘moderate’, ‘low’ and ‘Very Low’. Studies reporting each outcome were assessed across five domains: risk of bias, inconsistency, indirectness, imprecision and publication bias, with each rated as ‘not serious’, ‘serious’ or ‘very serious’. Each time a domain was rated as ‘serious’, the certainty score was downgraded by one level. If a domain was rated ‘very serious’, certainty was downgraded by two levels. Ratings across each domain were cumulative, such that if two domains were rated as ‘serious’, the overall certainty rating was downgraded two levels.

### Data synthesis and analysis

Outcome data from measures of mental health symptoms, work-related outcomes, user satisfaction, positive mental health, quality of life, help-seeking or adverse effects were extracted. Data were extracted from all measures, and data from multiple measures of the one outcome were both extracted. Data were also extracted at each follow-up point reported. Intention-to-treat data were extracted where available, and if not presented, per-protocol data were used. If studies did not report data of interest (ie, SEs instead of SD), the Cochrane recommended formula for calculating SD was applied.^32^

Quantitative data synthesis was performed using Comprehensive Meta-Analysis software V.3.^33^ Standardised mean differences (SMD; Cohen’s d), 95% Cls, and associated p values for outcome data were calculated and reported. A Cohen’s d of 0.2 was interpreted as a small effect, 0.5 a moderate effect and 0.8 a large effect.^34^ Effect sizes were deemed significant at the 0.05 level and were conducted using two-tailed tests. Separate random effect models, calculating a pooled SMD, were performed for each outcome of interest where there were at least two studies assessing that outcome. Heterogeneity was assessed using the Q and I^2^ statistics. Heterogeneity was confirmed if the Q statistic revealed a p<0.1, while I^2^ values of 25%, 50%, 75% represented low, moderate and substantial heterogeneity across studies, respectively. Funnel plots and the Egger test were used to assess publication bias. The fail-safe number was calculated for meta-analyses with significant effects, to determine how many additional studies with null findings would be required to increase the p value above the criterion of significance. Sensitivity analyses were planned on studies with low versus high risk of bias, as well as subgroup analyses on interesting grouping variables including type of feedback given after screening and by worker population.

## Results

**Figure 1:**
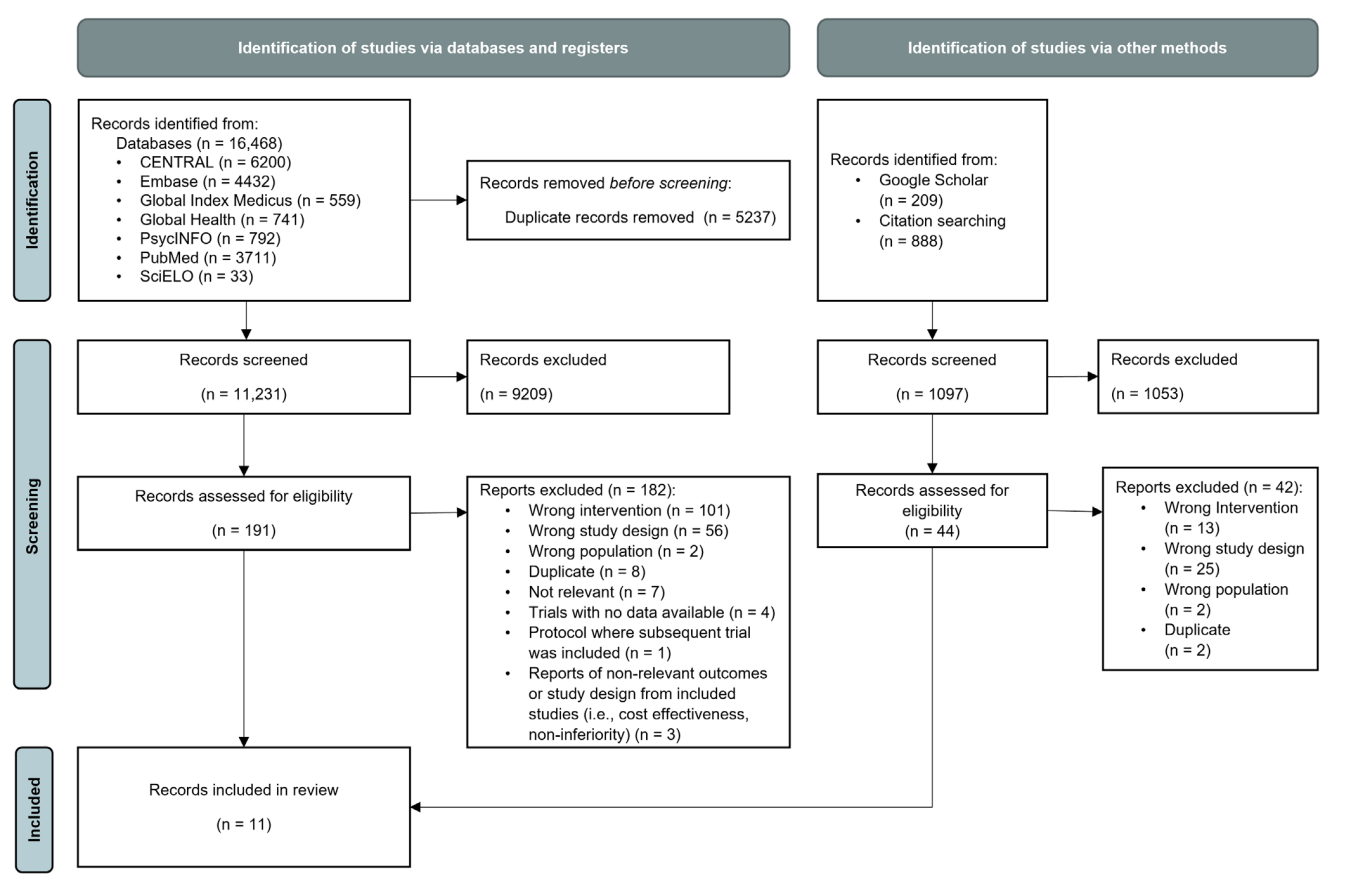
Study selection.

Database searching yielded a total of 11 231 records following duplicate removal (figure 1). Title and abstract screening resulted in the inclusion of 191 potentially relevant articles. After full-text screening, nine articles met inclusion criteria. Moderate inter-rater reliability was observed for full-text screening procedures (κ=0.65).^35^ Backward and forward citation searching revealed 2 additional articles that met inclusion criteria, yielding a total of 11 articles in this review. These 11 articles reported results from 8 independent trials. Notably, Klasen *et al* 36 reported long-term follow-up data from two independent trials also included.^37 38^ There was a three-arm RCT (The Mental Vitality @ Work Study) where two-intervention arms were compared with the same control group in two separate articles,^39 40^ and user satisfaction data were reported in an additional article.^41^ The remaining five trials were reported in single articles,^42–46^ one which was a three-arm trial reporting comparisons between all groups in the one article.^43^

Overall, data were reported from 2940 employees working in banking,^36–38^ healthcare,^39–41 46^ civil service,^43^ meat processing^42^ and mixed industries.^44 45^ All trials were undertaken in high-income countries, with four trials (six articles) conducted in The Netherlands, two in the USA, one in the UK and one in Belgium. All trials were RCTs, one of which was cluster randomised, one a cluster randomised stepped-wedge trial, and another using partial quasi-randomisation. Two studies were assessed as low risk of bias, one with some concerns and six with high risk of bias (online supplemental information 2).

10.1136/oemed-2022-108608.supp2 Supplementary data

Mental health screening tools identified were often study or occupational health service specific including the Balansmeter,^36–38^ Health Risk Appraisal^43^ or POSE.^42^ These assessed multiple health domains including mental health or stress. The WHS Module^39–41^ was also used (where participants completed screening using validated psychometric scales including the NWFQ, 4DSQ, etc). One trial using a health risk appraisal reported use of the Kessler Psychological Distress Scale followed by a more specific assessment of depression using the Quick Inventory of Depressive Symptomatology-Self Report.^45^ Farzanfar *et al* reported using the WHO-5 for screening purposes, while Steel *et al* administered a questionnaire-based screening algorithm using the 12-Item General Health Questionnaire (GHQ-12).^46^

Tables 1 and 2 describe the characteristics of included articles. Screening implementation varied considerably across studies, forming two distinct categories. First, six studies evaluated screening followed by feedback, advice and/or referral to treatment^37 40 42–44 46^ (table 1). In these studies, all participants completed screening, and intervention participants who screened positive were provided access to some form of follow-up health advice and/or consultation intervention with a health professional. Control participants completed screening but were not provided feedback or advice, however, could still access treatment-as-usual provided by their occupational health service or external providers if they wished. This applies except for Steel *et al*, who compared workers allocated to one of two active intervention arms who both received screening via different modalities. Here, screening as usual provided by an occupational physician was compared with more targeted online screening paired with referral and advice from an occupational physician for those most at risk of work-related illness.

**Table 1:** Characteristics of included studies evaluating screening followed by feedback, advice and/or referral

Study characteristics	Population	Screening tool; delivery	Screening process	Sample size; gender (% male); mean age (years)	Study design; measurement time points; intervention; control group	Advice description; delivery; adherence (% of participants)	Outcomes of interest (scale)	Efficacy of screening*/intervention
Kant *et al;*† The Netherlands in 2003^37^	Employees of large banking company (formally employed, not specified if full time/part time, mean work hours/week≈34).	Balansmeter, 34 items assessing multiple health domains including mental health; Delivered by occupational health service from participating company using posted questionnaire.	N=9863 invited to complete screening (completed by n=4950). Those screening ‘at-risk’ of sickness absence (n=299) (and who were not absent from work, not pregnant, not receiving treatment from the OP at the time of screening) were randomised.	IG: n=132 73.50% 46.32 CG: n=131 68.70% 46.58	RCT; Baseline, 1-year follow-up; Screening+consultation with OP; Screening+care as usual	Invited to OP consultation (1–1.5 hours) focusing on clarifying symptoms from screening, discussing benefits of treatment, and referral to targeted intervention); Delivery same as screening; 75%.	Sickness absence: duration, frequency, incidence of long-term absence (individual record linkage using company registers of certified sickness absence).	ITT analyses showed no significant intervention effect on sickness absence outcomes. Modified ITT (excluding those who had already sought treatment) revealed significant effect of intervention on sickness absence duration, but not frequency or incidence of long-term absence.
Klasen *et al;*†^36^	–	–	–	–	2-year, 3-year, 4-year and 5-year follow-up	–	Sickness absence: duration, frequency, incidence of long-term absence (individual record linkage using company registers of certified sickness absence). Work-related: termination of employment contract (company registers).	Kant *et al* 37 observed a marginally significant decrease in sickness absence duration and incidence of long-term absence at 5 years, no effect on frequency. No effect on termination of employment observed.
Gärtner *et al;*‡ The Netherlands in 2011^40^	Nurses/allied health professionals employed at an academic medical centre (formally employed, mix of temporary, part time and full time, mean work hours/week≈30).	WHS Mental Module screening for work functioning and mental health complaints; Delivered by the research team online.	Participants prerandomised according to cluster (ward). N=1152 invited, n=379 enrolled (completed baseline and were not expected to be on sick leave for >2 weeks at the study commencement). Once completed, intervention participants received automatically generated results and those screening positive (n=151) were invited to OP consult. Control participants did not receive screening results or any intervention.	IG: n=191 18.00% 43.00 CG: n=188 23.00% 42.00	Cluster RCT; baseline, 3-month, 6-month follow-ups; Screening and feedback+OP consultation; wait-list control.	Voluntary face-to-face OP consultation followed 7-step protocol similar to employee-initiated care-as-usual consultation but with a preventive focus; delivered by in-company occupational health service; 34%	Help-seeking: formal (visiting 1 of 11 caregivers). Mental health symptoms: psychological distress (4DKL), depression and anxiety (BSI), PTSD (IES). Work-related: impaired work functioning (NWFQ), need for recovery after work (VBBA).	Marginally significant effect of intervention on help-seeking at 3-month follow-up (stable in intervention, decline in control) attenuated at 6-month follow-up. No effect of intervention on mental health outcomes observed. Significant effect found for impaired work functioning favouring intervention at both 3 and 6 months. No effect on need for recovery after work.
Ketelaar *et al;*§ The Netherlands in 2011^41^	–	–	–	–	3-month and 6-month follow-ups.	–	User satisfaction (participant perspectives on screening including usefulness of advice/e-mental health programme provided, acceptability of feedback, perceived efficacy or the advice/e-mental health programme, and preferences regarding future screening).	41% (51 from 125) of participants from Gartner *et al* attended preventive consult, 51% (18 from 35) reported receiving advice, and 80% (16 of 20) reported following such advice. 13% (16 from 126) of participants reported wanting feedback differently. 60% (9 from 15) participants felt advice helped improve their mental health/work functioning. 37% (41 from 112) would appreciate to be offered the screening intervention in the future.
Farzanfar *et al*; United States date not specified^44^	Employed individuals from various industries/workplaces (type of employment not specified).	WHO-5 and 1-item from PHQ; delivered by the research team via telephone.	N=463 volunteered to participate. Those screening positive (n=164) for emotional distress (WHO-5) and functional impairment (PHQ), who were not currently undertaking mental health treatment, were randomised. Control participants were advised to consult their usual care provider if required.	IG: n=87 27.00% 39.00 CG: n=77 21.80% 39.20	RCT; baseline, 3, 6 months; Screening+TLC-Detect (all modules); screening only+TLC-detect (screening only).	Automated phone intervention with three modules: (1) screening, (2) intervention, containing education and referral for self-help or professional care tailored to symptoms, and (3) follow-up, involving monthly calls to monitor progress and adherence; delivered via algorithm on the phone; 78% responded to follow-up call.	Mental health symptoms: depression (PHQ-9), stress (PSS). Positive mental health: well-being (WHO-5). QoL and functioning: Mental and physical well-being (SF-12). Work-related: productivity, absenteeism, presenteeism (Work Limitation Questionnaire). User satisfaction: usability (HTQ).	Significant intervention effect on depression at 3 months, attenuated at 6 months. No effect on stress or well-being observed. No significant effect on QoL at 3 months, but effect observed at 6 months. No effect of intervention on work-related outcomes, except for time/scheduling at 6 months and interpersonal scale at 3 months. 84% intervention participants reported ease of use and 76% said intervention was informative. Not assessed in control.
Addley *et al* (2014);¶ UK in 2009^43^	Administrative and executive grade employees within civil service (formally employed, 87% full time).	Health risk appraisal (‘LPAA’) assessing multiple health domains including stress; delivered by the company occupational health service.	N=1503 invited to complete baseline questionnaire. From the n=334 who completed, n=180 were randomised. Those allocated to intervention completed the LPAA, which provides a printout of an individual’s health risk profile and general advice. Those allocated to control received no LPAA or subsequent intervention.	IG—group B: n=43 54.00% 60% aged 25–44 CG: n=48 46.00% 50% aged 25–44	RCT; Baseline and 12 months; IG-group B: screening; No intervention.	IG—group B received the 45 min HRA assessment only; Delivered by the company occupational health service and company sport organisation; Not reported.	Mental health symptoms: psychological distress (GHQ-12). Positive mental health: well-being (WHO-5 and MHC-SF). Work-related outcomes: work ability (WAI) and job satisfaction (single item).	No effect of either intervention on any outcome variables.
van Holland *et al*; The Netherlands in 2012^42^	Meat processing plant employees (formally employed for at minimum 12 hours/week).	POSE programme assessing multiple health domains including mental health; Delivered part online part face to face in the workplace by a registered vocational physiotherapist.	Participants prerandomised by cluster (workplace). N=986 invited. Intervention participants agreeing to participate completed screening tests to create a risk profile.	IG: n=305 89% 50.60 CG: same as IG (see trial design)	Cluster randomised stepped wedge trial; baseline, 1–3 years follow-up (depending on wedge); POSE (screening+counselling/referral); care as usual.	Built into screening procedures (as part of POSE) was a counselling session regarding participant results. Based on scores, participants received recommendations on future interventions (ie, consulting a GP, physio, etc); Not reported.	Positive mental health: vitality (RAND-36). QoL and functioning: self-rated health (EuroQol-5D). Work-related: sickness absence (company data), work ability (WAI), productivity (QQ), psychosocial work factors (COPSOQ II).	No effect of intervention found on any outcomes. Significantly higher odds of longer-term sickness absence and lower productivity in intervention vs control at follow-up.
Steel et al;** Belgium in 2019^46^	Hospital workers in roles requiring safety functions, heightened vigilance, work involving physical, biological, or chemical agents or tasks that are ergonomically or mentally burdensome (52.6% medical personnel).	Physical assessment and OP consultation or online health screening questionnaire (GHQ-12 and other measures of work-related illness); OP consultation conducted face to face, screening questionnaire conducted online.	Participants allocated to one of four groups (randomly allocated and non-random allocated IG and randomly allocated and non-randomly allocated CG). IG participants completed online health screening questionnaire and the top 20% of participants reporting symptoms most indicate of needing OP contact were targeted and referred for OP consultation to discuss results. The other 80% did not receive further care. CG participants were invited to attend health screening with an OP consultation.	IG: n=380 17.1% 45.10 CG: n=396 19.7% 45.63	RCT trial with quasi- randomisation; baseline, 6-month and 18-month follow-ups; random and non-random IG and GC.	OP could refer participants on to other healthcare providers depending on their presenting issue; OP was employed by hospital site; All CG participants received OP consult but % taking up subsequent referral not stated, all IG participants received online health screening and N=126 were referred to OP but N/% taking up OP consult, or any subsequent referral not stated††.	Mental health symptoms: stress and burnout (COPSOQ-III), general mental health (GHQ-12) Work-related: absenteeism and presenteeism (iPCQ), need for recovery after work (NFR), role conflicts and job satisfaction (COPSOQ-III), turnover intention (VBBA) User satisfaction: trust in OP (Trust in Physician Scale) QoL and functioning: self-perceived health (EQ-5D) Help-seeking: spontaneous consultations with OP	No overall group differences between groups in GHQ-12 but reduced general mental health for IG at 18-month follow-up. Non inferiority between groups reported for GHQ-12 and stress. No overall difference in turnover intention, but IG had fewer absences not observed at follow-up. Superior post hoc differences favouring targeted screening in absenteeism. Non inferiority between groups reported for absenteeism, presenteeism, need for recovery, and job satisfaction. IG reported lower trust in OP overall. No overall difference between groups for EQ-5D. Non inferiority reported. IG had fewer spontaneous consultations with OP overall not sustained at 18-month follow-up. Superior post hoc differences favouring CG in spontaneous consultations.

*A significant intervention effect refers to instances where a study reported a significant difference in a particular outcome between intervention and control groups comparing preintervention and postintervention scores on that outcome.

†Klasen *et al* 36 reports results of Kant *et al* 37 and Lexis *et al* 38 (see table 2) at 2–5 years follow-up (in 1-year increments).

‡Gärtner *et al* 40 and Boiler *et al* 39 (see table 2) report revaluations from the one, three-armed trial ‘Mental Vitality @ Work’ trial. As such, the control groups between these two studies are the same.

§Ketelaar *et al* 41 report additional data from the intervention groups reported in Gärtner *et al* 40 and Boiler *et al*.^39^

¶Three-arm trial comparing two interventions to control, both reported in the one paper. Results from other intervention arm are reported in table 2. Note the Cochrane recommended formula for calculating SD from SEs was applied to extracted quantitative data from this study.

**Note this article was identified via updated database searching and follow-up data collection occurred during the COVID-19 pandemic.

††Note number of referrals measured and means for whole sample at each survey wave reported, however, N/% of referrals not reported.

BSI, brief symptom inventory; CG, control group; COPSOQ II, Copenhagen Psychosocial Questionnaire; 4DKL, Four-Dimensional Symptoms Questionnaire; EQ-5D, EuroQol 5-dimension Health-Related Quality of Life Instrument; GHQ-12, 12-Item General Health Questionnaire; HTQ, Health Technology Questionnaire; IES, Impact of Events Scale; IG, intervention group; iPCQ, IMTA Productivity Cost Questionnaire; ITT, intention to treat; LPAA, Lifestyle and Physical Activity Assessment; MHC-SF, Mental Health Continuum Short Form; NFR, Need for Recovery Scale; NWFQ, Nurses Work Functioning Questionnaire; OP, occupational physician; PHQ, Patient Health Questionnaire; POSE, Promotion of Sustained Employability; PSS, Perceived Stress Scale; QQ, Quality and Quantity Questionnaire; RCT, randomised controlled trial; SF-12, 12 Item Short Form Survey; VBBA, Questionnaire on the Experience and Evaluation of Work; WAI, Work Ability Index; WHS, Work Health Surveillance.

**Table 2:** Characteristics of included studies evaluating screening followed by feedback, advice and/or allocation to treatment

Study characteristics	Population	Screening tool; delivery	Screening process	Sample size; gender (% male); mean age (years)	Study design; measurement time points; intervention; control group	Treatment description; delivery; adherence (% of participants)	Outcomes of interest (scale)	Efficacy of screening*/intervention
Lexis *et al*;† The Netherlands in 2007^38^	Employees of large banking company (formally employed, not specified if full time/part time, mean work hours/week≈35).	Balansmeter, 34 items assessing multiple health domains including mental health; Delivered by occupational health service from participating company using posted questionnaire.	N=23 973 invited to complete screening (completed by n=9157). Those screening ‘at-risk’ of sickness absence and reporting mild-severe depression symptoms on the HAD-D (n=211) (and who were not absent from work, not pregnant, not receiving treatment at the time of screening) were invited to complete a baseline questionnaire (assessing BDI-II among others). Eligible responders (n=139) were randomised.	IG: n=69, 60.90% 48.41 CG: n=70 61.40% 47.07	RCT; Baseline, 6-month, 12-month and 18‡-month follow-ups; screening+psychological treatment; screening+care as usual	Individual psychological treatment (PST and CBT). Delivered face-to-face in seven standardised 45 min sessions (+up to five additional sessions if needed); delivered by external psychologists that regularly provided psychological healthcare for the company; 55%.	Mental health symptoms: depression (HADS-D, BDI-II), psychological distress (BSI). Sickness absence: duration, frequency, incidence of long-term absence (individual record linkage using company registers of certified sickness absence). Quality of life and functioning: self-rated health (SF-36). Work-related: job demands, decision latitude, social support (JCQ), job insecurity (VBBA).	Significant intervention effect in decreasing depression (both HADS-D and BDI-II) and psychological distress at both 6 months and 12 months. Significant intervention effect on sickness absence duration at 12 months, but not 18 months. No effect on frequency or long-term absence. No effect observed for work-related variables (job demands, decision latitude, social support, job insecurity) nor for self-rated health.
Klasen *et al*†^36^	–	–	–	–	2-year, 3-year, 4-year and 5-year follow-up	–	Sickness absence: duration, frequency, incidence of long-term absence (individual record linkage using company registers of certified sickness absence). Work-related: termination of employment contract (company registers).	Lexis *et al* 38 observed no effect on sickness absence duration, frequency, or risk of long-term absence at 5 years follow-up. A significant effect favouring intervention observed for termination of employment.
Bolier *et al;*§ The Netherlands in 2011^39^	Nurses/allied health professionals employed at an academic medical centre (formally employed, mix of temporary, part time and full time, mean work hours/week≈31).	WHS Mental Module screening for work functioning and mental health complaints; Delivered by the research team online.	Participants prerandomised according to cluster (ward). N=1140 invited, n=366 enrolled (completed baseline and were not expected to be on sick leave for >2 weeks at the study commencement). Once completed, intervention participants received automatically generated results and access to intervention. Control participants did not receive screening results or any intervention.	IG: n=178 17.40% 38.00 CG: n=188 22.90% 42.00	Cluster RCT; baseline, 3-month, 6-month follow-ups; Screening and feedback+online intervention; wait-list control.	Participants offered choice of online self-help interventions tailored to reported symptoms, accessed via a website with a log in; delivered online by the research team; 5%	Mental health symptoms: depression and anxiety (BSI). Positive mental health: positive mental health (MHC-SF), well-being (WHO-5). Work-related outcomes: work engagement (UWES-9).	No effect of intervention on depression, anxiety or well-being. Significant effect of intervention on positive mental health at 3 months and 6 months observed. Significant effect of intervention on work engagement (decline in control group).
Ketelaar *et al;*¶ The Netherlands in 2011^41^	–	–	–	–	3-month and 6-month follow-ups.	–	User satisfaction (participant perspectives on screening including usefulness of advice/e-mental health programme provided, acceptability of feedback, perceived efficacy or the advice/e-mental health programme, and preferences regarding future screening).	5% of participants from Boiler *et al* started the e-health interventions. 17% (14 from 82) of participants reported wanting feedback differently. 0% (0 from 4) participants felt following e-mental health intervention helped improve their mental health/work functioning. 33% (23 from 69) would appreciate to be periodically offered the screening intervention in the future.
Wang *et al*; USA in 2004^45^	Workers from various large companies/industries enrolled in a behavioural healthcare provider (formally employed full-time mean work hours/week≈42).	Two-phase screening: (1) Health Risk Appraisal (screening for psychological distress using K6) followed by (2) more specific assessment of depression using the QIDS-SR; Delivered by the behavioural healthcare company via telephone.	Phase (1) invited N=113 843 via targeted email, N≈150 000 via company-wide email, and N=1 14 635 via healthcare member mail survey. Those interested and screening positive with healthcare coverage (n=7978) were invited via letter/telephone to participate in phase (2). In phase (2), those screening positive (without history of mania, substance dependence, suicidal ideation/attempts, or specialist mental health treatment in the prior year) were randomised.	IG: n=304 29.30% 40.70 CG: n=300 23.00% 42.40	RCT; baseline, 6, 12 months; screening+telephone intervention programme; screening+care as usual.	Intervention involved assessment, facilitated appropriate in-person treatment (ie, psychotherapy/medication), and supported treatment adherence. Those declining in-person treatment offered structured psychotherapy intervention via telephone. All sent psychoeducational workbook; Delivered by mental health professionals employed by the healthcare provider who received training/supervision from research team; 90% completed initial care management contact.	Mental health symptoms: depression (QIDS-SR) Work-related: productivity including hours, performance, turnover, incidents (HPQ)	Significant intervention effect at 6 months and 12 months on decrease in depression severity. Significant intervention effect at 12 months on proportion of participants with improved symptoms/recovery. Significant intervention effect at 6 months and 12 months on effective hours worked and role retention. No effect on actual hours worked, job performance or incidents.
Addley et al;** UK in 2009^43^	Administrative and executive grade employees within civil service (formally employed, 87% full time).	Health risk appraisal (‘LPAA’) assessing multiple health domains including stress; delivered by the company occupational health service.	N=1503 invited to complete baseline questionnaire. From the n=334 who completed, n=180 were randomised. Those allocated to intervention completed the LPAA, which provides a printout of an individual’s health risk profile and general advice. Those allocated to control received no LPAA or subsequent intervention.	IG—group A: n=41 49.00% 51% aged 25–44 CG: n=48 46.00% 50% aged 25–44	RCT; baseline and 12 months; IG—group A: screening+healthworks; no intervention	IG—group A received 45 min HRA assessment, feedback, ‘Healthworks’ half-day health and well-being education programme, access to lifestyle modules, and up to 12 months of health coaching; Delivered by the company occupational health service and company sport organisation; all attended healthworks programme	Mental health symptoms: psychological distress (GHQ-12). Positive mental health: well-being (WHO-5 and MHC-SF). Work-related outcomes: work ability (WAI) and job satisfaction (single item).	No effect of either intervention on any outcome variables.

*A significant intervention effect refers to instances where a study reported a significant difference in a particular outcome between intervention and control groups comparing preintervention and postintervention scores on that outcome.

†Klasen *et al* 36 reports results of (Kant *et al* 37 (see table 1) and Lexis *et al* 38 at 2–5 years follow-up (in 1-year increments).

‡Sickness absence only data for 12-month and 18-month follow-up only.

§Gärtner *et al* 40 (see table 1) and Boiler *et al* 39 report revaluations from the one, three-armed trial ‘Mental Vitality @ Work’ trial. As such, the control groups between these two studies are the same.

¶Ketelaar *et al* 41 report additional data from the intervention groups reported in Gärtner *et al* 40 and Boiler *et al.* 39

**Three-arm trial comparing two interventions to control, both reported in the one paper. Results from other intervention arm are reported in table 1. Note the Cochrane recommended formula for calculating SD from SEs was applied to extracted quantitative data from this study.

BDI-II, Beck Depression Inventory; BSI, Brief Symptom Inventory; CBT, cognitive–behavioural therapy; CG, control group; GHQ-12, General Health Questionnaire; HADS, Hospital Anxiety and Depression Scale; HPQ, Health and Productivity Questionnaire; IG, intervention group; JCQ, Job Content Questionnaire; K6, Kessler Psychological Distress Scale; LPAA, Lifestyle and Physical Activity Assessment; MHC-SF, Mental Health Continuum Short Form; PST, problem-solving therapy; QIDS-SR, Quick Inventory of Depressive Symptomatology-Self Report; RCT, randomised controlled trial; SF-36, 36 Item Short Form Survey; UWES-9, The Utrecht Work Engagement Scale; VBBA, Questionnaire on the Experience and Evaluation of Work; WAI, Work Ability Index; WHS, Work Health Surveillance.

Second, four studies evaluated screening followed by feedback, advice and allocation to treatment^38 39 43 45^ (table 2). In these latter ‘mixed-intervention’ studies, participants in the intervention group were provided treatments that those in the control group could not access. Types of treatment identified included individual treatment with a psychologist,^38^ online interventions tailored to symptom profiles,^39^ telephone care management and psychotherapy with ongoing treatment adherence monitoring or phone-based psychotherapy,^45^ or health and well-being educational/coaching sessions.^43^ Uptake of these interventions varied considerably at 5% for online self-help interventions,^39^ 55% for individual psychotherapy,^38^ 90% for care management^45^ and 100% for educational sessions.^43^

Given this distinction between screening followed by advice or treatment, and that the primary aim of this review was to disentangle the efficacy of screening in improving mental health symptoms and other outcomes, a decision was made to analyse the efficacy of advice and mixed-intervention treatment studies separately. Further, only comparisons in studies where the control group did not receive any advice/intervention beyond the screening questionnaire(s) were quantitatively analysed. Because of the small number of studies and sparsity of data available across multiple measures (ie, depression, psychological distress) of each outcome (ie, mental health symptoms) at different follow-up periods (ie, 3–12 months), the pooled intervention effect size combining measures using the longest follow-up time point of each study was calculated. However, individual study specific results of all measures associated with outcomes of interest are presented in tables 1 and 2.

**Figure 2:**
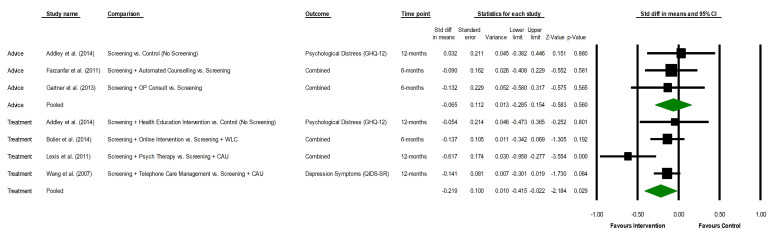
Forest plots of the effects of screening followed by advice and screening followed by treatment on employee reported mental health symptoms. CAU, Care as Usual; GHQ-12, 12-Item General Health Questionnaire; QIDS-SR, Quick Inventory of Depressive Symptomatology-Self Report; WLC, Wait List Control.

The forest plots depicting intervention effects on mental health symptoms are provided in figure 2. As shown, the pooled SMD between the intervention and control groups comparing change from baseline and longest follow-up was d=−0.07 (95% CI −0.29 to 0.15), indicating a null effect of screening followed by advice on employee reported mental health outcomes (table 3). No evidence of heterogeneity was observed (Q=0.32; p=0.853). Neither funnel plot nor Egger’s test of the intercept (t=0.13; p=0.915) demonstrated any evidence of publication bias. Combining the four studies evaluating screening followed by treatment resulted in a pooled SMD of d=−0.22 (95% CI −0.42 to −0.02) indicating a reduction in mental health symptoms favouring the intervention. Moderate, but non-significant, heterogeneity was detected in this analysis (Q=7.07; p=0.070). Publication bias was not observed (t=0.70; p=0.558; visual inspection of plot) and the fail-safe number was nine. Although sensitivity analysis by risk of bias and subgroup analyses and were planned a priori, these were not performed given the small number of eligible studies identified.

**Table 3:** Meta-analysis results describing pooled effect of workplace mental health screening followed by advice or treatment on outcomes of interest

		N studies	Cohen’s d (95% CI)	P value	Heterogeneity (I^2^)	Certainty rating (GRADE)*
Screening+advice	Mental health symptoms (combined)	3	−0.07 (–0.29 to 0.15)	0.560	0	⊕⊕ΟΟ LOW†‡
	Work-related (sickness absence duration)	2	0.06 (–0.22 to 0.34)	0.675	75.88	⊕ΟΟΟ VERY LOW†‡§
	Work-related (impairment)	2	−0.27 (–0.49 to –0.05)	0.018	0	⊕ΟΟΟ VERY LOW†‡§¶
	Work-related (positive)	2	−0.03 (–0.42 to 0.36)	0.881	68.81	
	User satisfaction	2	–	–	–	⊕ΟΟΟ VERY LOW†‡¶
	Positive mental health (combined)	2	0.06 (–0.20 to 0.31)	0.659	0	⊕⊕ΟΟ LOW†‡
	QoL and functioning	1	Not reported	–	–	⊕ΟΟΟ VERY LOW†‡
	Help-seeking**	1	−0.18 (–0.49 to 0.13)	0.250	–	⊕⊕ΟΟ LOW‡¶
	Adverse effects	0	–	–	–	–
Screening+treatment	Mental health symptoms (combined)		−0.22 (–0.42 to –0.02)	0.029	57.59	⊕⊕ΟΟ LOW†§
	Work-related (sickness absence duration)††	1	−0.38 (–0.71 to –0.04)	0.029	–	⊕ΟΟΟ VERY LOW†‡
	Work-related (impairment)	0	–	–	–	–
	Work-related (positive)	3	0.24 (–0.04 to 0.52)	0.093	75.62	⊕ΟΟΟ VERY LOW†‡§¶
	User satisfaction	1	–	–	–	⊕ΟΟΟ VERY LOW‡¶
	Positive mental health (combined)	2	0.14 (–0.04 to 0.33)	0.135	0	⊕⊕ΟΟ LOW†‡
	QoL and functioning	1	Not reported	–	–	⊕ΟΟΟ VERY LOW†‡
	Help-seeking	0	–	–	–	–
	Adverse effects	0	–	–	–	–

*GRADE Levels of Certainty: HIGH ⨁⨁⨁⨁: Very confident that the true effect lies close to that of the estimate of the effect. MODERATE ⨁⨁⨁◯: Moderately confident in the effect estimate: the true effect is likely to be close to the estimate of the effect, but there is a possibility that it is substantially different. LOW ⨁⨁◯◯: Confidence in the effect estimate is limited: the true effect may be substantially different from the estimate of the effect. VERY LOW ⨁◯◯◯: Very little confidence in the effect estimate: the true effect is likely to be substantially different from the estimate of effect.

†Downgraded due to risk of bias.

‡Downgraded due to imprecision.

§Downgraded due to inconsistency.

¶Downgraded due to indirectness.

**Effect size at 6-month follow-up tabulated, however, at 3 months, d=0.32 (0.02–0.62); p=0.036.

††12 months data reported. Effect attenuated at 5-year follow-up, d=0.11 (−0.34 to 0.55); p=0.634. Longest follow-up period reported by each study contributed to meta-analysis across all outcomes. Outcomes with only one study were not meta-analysed, but raw data were extracted and converted into a standardised mean difference (Cohen’s d) from that single study.

GRADE, Grading of Recommendations Assessment, Development and Evaluation.

The pooled effect of screening followed by advice on sickness absence duration up to 12 months follow-up was not significant (d=0.06 (95% CI −0.22 to 0.34)). One of these trials reported a marginally significant difference in mean days of sickness absence at long-term follow-up (5 years) favouring the intervention (see Klasen *et al* 36). Only one study evaluating screening followed by treatment assessed sickness absence,^38^ indicating a significant reduction in sickness absence favouring the intervention at 12 months (d=−0.38 (95% CI −0.71 to −0.04)) that was attenuated at 5-year follow-up.^36^ Pooled analysis of impaired work functioning found a small and significant decrease (ie, better work functioning) favouring screening followed by advice (d=−0.27 (95% CI −0.49 to −0.05)) at 6-month follow-up. However, screening followed by advice appeared less effective when pooling positively balanced work-related outcomes (including work performance and satisfaction; d=−0.03 (95% CI −0.42 to 0.36)). Finally, the pooled intervention effect for screening plus treatment studies on positively balanced work-related outcomes (including productivity, job satisfaction, work ability and engagement) showed a small but not significant effect favouring intervention (d=0.24 (95% CI −0.04 to 0.52)).

User satisfaction was not compared between intervention and control groups in any trial. Farzanfar *et al* 44 reported that 76% of intervention participants found screening informative, 65% found it very or somewhat useful, and 47% agreed that the system reduced their visit time with their doctor.^44^ Additionally, Ketelaar *et al* found that 60% of participants (n=9) from Gartner *et al* who received advice from an occupational physician claimed that it helped improve their mental health/work functioning, and 37% (n=41) would appreciate being offered the screening intervention in the future. No participants (0 from 4) from Boiler *et al* reported that the e-mental health treatment intervention helped improve their mental health/work functioning, while only 33% (n=23) indicated appreciation of the screening intervention in the future. Another trial found that those receiving face-to-face screening and consultation with a physician reported greater trust in that physician compared with those who completed targeted screening online.^46^

Neither screening followed by advice (d=0.06; 95% CI −0.20 to 0.31)) or screening followed by treatment (d=0.14 (95% CI −0.04 to 0.33)) improved employee reported positive mental health at longest reported follow-up. Similar null effect for quality of life were observed. Finally for help-seeking, only one trial evaluating screening followed by advice reported a significant intervention effect at 3-month follow-up (d=0.32; (95% CI 0.02 to 0.62)) that was no longer observed at 6 months (d=−0.18 (95% CI −0.49 to 0.13)).^40^ No study reported instances of participant reported harms and adherence rates are reported in tables 1 and 2 for each study.

There was a low level of certainty about the lack of effect of screening followed by advice on employee mental health symptoms (table 3; online supplemental information 3). Similarly, the small positive impact of screening followed by treatment on employee mental health symptoms was rated with low certainty. Certainty ratings for all other outcomes for both screening approaches were either low or very low.

10.1136/oemed-2022-108608.supp3 Supplementary data

## Discussion

While mental health screening is increasingly implemented in workplaces, this review identified surprisingly few controlled trials evaluating the efficacy of this intervention. Critically, this review identified 8 trials (reported across 11 studies) and observed considerable variation in screening implementation. The pooled effect of the available data showed that screening augmented with feedback and advice had no detectable effect on employee mental health symptoms. Results from studies evaluating screening augmented with allocation to specific treatment interventions showed a small positive effect in reducing employee reported mental health symptoms, though with only a low level of certainty regarding this effect. Together these results suggest that direct referral to access postscreening treatment interventions can add value to the process of screening as an approach to reduce mental health symptoms in workers. However, this is not observed when only providing screened individuals with information about what referral options are available. This review also identified extremely limited data on the effect of workplace mental health screening on work-related outcomes and the few trials that recorded user-satisfaction outcomes produced mixed results.

The key finding from this study is that mental health screening programmes, when paired with feedback and advice but not direct access to new or different treatment programmes, does not lead to improvements in employee mental health or reduce sickness absence. This is a challenging finding as mental health screening and advice as a stand-alone approach (not paired with direct access to an intervention) is increasingly implemented in workplaces. One explanation for the apparent lack of effect for mental health screening is a low adherence to the advice offered to workers in the absence of direct access to a specific treatment. Trials assessing this screening implementation method often did not comprehensively assess or report the type and extent of advice provided to participants, nor what proportion of participants went on to receive referred treatments. One trial evaluating screening followed by advice, and/or referral noted that approximately one in six participants followed an intervention after screening.^42^ Another reported that 151 employees screened positive, but only 51 visited their occupational physician.^40^ Of these, only 18 reported having received advice.^41^ Overall, while this approach aims to show workers experiencing mental health symptoms where they can seek help, it concurrently places the onus on the individual worker to action the advice received. Barriers preventing individuals accessing treatment such as stigma or availability and cost^47 48^ may remain, in turn, preventing individuals to action referrals provided. Providing direct access to relevant interventions/treatments may overcome this.

This review did find low certainty evidence for a small beneficial effect of screening augmented with access to new treatments. Cognitive–behavioural therapy (CBT)-based interventions have been effective at reducing employee mental health symptoms in previous reports.^14^ This review observed similar findings in that the two trials incorporating CBT components produced the strongest effects on employee mental health outcomes.^38 45^ While a pooled effect size of d=0.2 may be small, existing research on workplace interventions aimed at preventing depression report similar sized effects.^15^ Even a small improvement in employee reported mental health symptoms may have an important impact on the performance and functioning of employees as well as an organisation, particularly where individuals are not reporting clinical levels of symptoms. However, whether screening adds to this intervention effect, over and above what may have been observed if these CBT-based interventions were offered to any worker who felt they may benefit, remains unclear.

It is also worth considering how the effect of screening augmented with an intervention like CBT compares to other workplace mental health interventions. Evidence shows that interventions targeting the work environment and broader organisational systems such as flexible work arrangements,^49^ participatory interventions targeting job design,^50^ and improvements to workload and scheduling^51^ are associated with improvements in mental health symptoms. Further, manager mental health training has been found to improve managers’ attitudes towards mental health and their application of behaviours to better support the mental health of their staff.^52–55^ Information obtained via mental health screening may highlight where to direct evidence based interventions (eg, in specific units/departments that may have higher symptom levels) that may, in turn, improve mental health outcomes. However, the accuracy of workplace mental health screening may limit the utility of this approach. Here, confidentiality is a key consideration for many workers, and if not adequately addressed, may motivate under-reporting of symptoms.^25^

The evidence included in this review has several limitations. First, the level of confidentiality for workers screening responses in the trials identified in this review was difficult to determine. Many of the included trials used screening administered by occupational health services of participating organisations, which may be interpreted by employees as an employer-administered survey. Therefore, included data may be prone to a self-censoring bias and potentially attenuated reporting because of employees’ perception that their employers will be able to access their responses. Indeed, one included study found that only 5% of participants reported not knowing (or not having met previously) the occupational physician conducting screening related consultations, yet around 50% had known them for 5 years or more.^46^ Second, the type of feedback and advice provided to participants was not clear. Whether this feedback was generic or tailored to participants’ screening responses may have important implications on their propensity to action the feedback received. Further research examining how best to provide feedback to create behaviour change conducive to improving mental health is warranted. Additionally, only included two trials included underwent cost-effectiveness analysis, one indicating favourable results^56 57^ and another indicating no cost–benefit.^42^ Future work in both research and industry should examine if the negligible benefits of screening are worth the costs of implementation.

The included trials also evaluated screening as a one-off intervention with relatively short-term follow-up measurement points ranging between 6 months and 12 months. It may be that study measurement time points informing this result may not have been long enough for changes in mental health symptoms to be observable. For example, mental healthcare shortages may delay engagement with professional care referred during screening. It is important to note here, however, that two trials did provide 5-year follow-up sickness absence data, indicating negligible benefits of both screening followed by advice and by treatment.^36^ User satisfaction with the screening programmes yielded mixed results, which somewhat opposed the wider literature reporting confidentiality issues when screening for mental health at work. Finally, the overall body of research identified is very diverse, with screening approaches, intervention delivery and assessed outcomes varying between trials.

The review process used was also not without limitations. First, given the small body of research identified, the meta-analyses conducted produced imprecise pooled estimates with wide CIs. Performing meta-analyses on a small body of studies has the potential to inflate the results generated. This review was also unable to perform sensitivity analyses by risk of bias, which limited the certainty of our findings as many trials were at risk of bias. Second, unpublished data from screening trials not registered on CENTRAL were not considered in this review. A systematic review investigating workplace suicide prevention reports that many studies in such area are not reported in academic literature.^58^ It is plausible that this may be the case here given widespread use of screening in industry. Finally, this review excluded trials involving military populations. While it may not be appropriate to extrapolate the findings from military-based screening onto general worker populations, much of the high-quality research in this area has been performed in such population. However, it is worth noting that our results finding that screening alone does not benefit mental health, aligns with that of the largest study of mental health screening in the military.^28^

## Conclusion

In summary, the currently available evidence suggests that unless workplace mental health screening is paired with access to postscreening treatment interventions, there is no indication that screening alone improves employee mental health. Interventions that prevent and promote mental health in the workplace are an important public health priority. This review serves as a call to action for further comprehensive evaluation of screening and other common workplace interventions aimed at improving the mental health of employees.
