# Supplementary material for: Workplace mental health screening: a systematic review and meta-analysis
Source: Occup Environ Med. 2023 Jun 15;80(8):469–84. doi: 10.1136/oemed-2022-108608 (PMC10423530; doi:10.1136/oemed-2022-108608)
Supplement: Supplementary data [file oemed-2022-108608supp001.pdf]

Database	Population AND	Intervention AND	Study Design/Control AND	Outcomes
**PubMed**	("Occupational Groups"[Mesh] OR "Occupational Health"[Mesh] OR "enterprise*"[tiab] OR<br>"business*"[tiab] OR<br>"employed"[tiab] OR<br>"employee*"[tiab] OR<br>"employer*"[tiab] OR<br>"employment*"[tiab] OR<br>"informal sector*"[tiab] OR<br>"informal work*"[tiab] OR<br>"laborer*"[tiab] OR<br>"labourer*"[tiab] OR<br>"occupation*"[tiab] OR<br>"personnel*"[tiab] OR<br>"professional"[tiab] OR<br>"professionals"[tiab] OR<br>"staff"[tiab] OR<br>"staffing*"[tiab] OR<br>"vocation"[tiab] OR<br>"vocations"[tiab] OR<br>"worker*"[tiab] OR<br>"workforce"[tiab] OR<br>"workplace*"[tiab] OR<br>"work place*"[tiab] OR<br>"worksite*"[tiab]<br><br>OR<br><br>"ambulance*"[tiab] OR<br>"paramedic*"[tiab] OR<br>"disaster responder*"[tiab] OR<br>"doctor*"[tiab] OR<br>"emergency service*"[tiab] OR<br>"emergency responder*"[tiab] OR<br>"emergency medical"[tiab] OR<br>"firefighter*"[tiab] OR<br>"fire fighter*"[tiab] OR<br>"first responder*"[tiab] OR<br>"general practitioner*"[tiab] OR	(screen*[tiab] OR<br>monitoring[tiab] OR<br>check[tiab] OR<br>check-up[tiab] OR<br>surveillance[tiab] OR<br>detection[tiab])<br><br>AND<br><br>("employee*"[tiab] OR<br>"laborer*"[tiab] OR<br>"labourer*"[tiab] OR<br>"personnel*"[tiab] OR<br>"professionals"[tiab] OR<br>"staff"[tiab] OR<br>"worker*"[tiab] OR<br>"workforce"[tiab] OR<br>"workplace*"[tiab] OR<br>"work place*"[tiab] OR<br>"worksite*"[tiab])	"Randomized Controlled Trial"[Publication Type] OR<br>"Randomized Controlled Trials as Topic"[Mesh] OR<br>"Non-Randomized Controlled Trials as Topic"[Mesh] OR<br>"Random Allocation"[Mesh] OR<br>"RCT"[tiab] OR<br>"randomized controlled trial"[tiab] OR<br>"randomised controlled trial"[tiab] OR<br>"non-randomized controlled trial"[tiab] OR<br>"non-randomised controlled trial"[tiab] OR<br>"non randomized controlled trial"[tiab] OR<br>"non randomised controlled trial"[tiab] OR<br>"random allocation"[tiab] OR<br>"random assignment"[tiab] OR<br>"randomized"[tiab] OR<br>"randomised"[tiab] OR<br>"quasi-experiment*"[tiab] OR<br>"quasi experiment*"[tiab] OR<br>"control group"[tiab] OR<br>"comparison group"[tiab] OR<br>"wait-list control"[tiab] OR<br>"wait list control"[tiab] OR<br>"control condition"[tiab]	"Mental Disorders"[Mesh] OR<br>"Mental Health"[Mesh] OR<br>"Psychology, Industrial"[Mesh] OR<br>"Stress, Psychological"[Mesh] OR<br>"adjustment"[tiab] OR<br>"affective disorder*"[tiab] OR<br>"anxiet*"[tiab] OR<br>"bipolar*"[tiab] OR<br>"burn out*"[tiab] OR<br>"burnout*"[tiab] OR<br>"CMD" [tiab] OR<br>"depressi*"[tiab] OR<br>"eating disorder*"[tiab] OR<br>"mental disorder*"[tiab] OR<br>"mental health*"[tiab] OR<br>"mental illness*"[tiab] OR<br>"mood disorder*"[tiab] OR<br>"obsessive compulsive disorder*"[tiab] OR<br>"ocd"[tiab] OR<br>"panic disorder*"[tiab] OR<br>"phobi*"[tiab] OR<br>"post traumatic*"[tiab] OR<br>"psychiatric diagnos*"[tiab] OR<br>"psychiatric disease*"[tiab] OR<br>"psychiatric disorder*"[tiab] OR<br>"psychiatric illness*"[tiab] OR<br>"psychological disorder*"[tiab] OR<br>"psychos*"[tiab] OR<br>"psychotic*"[tiab] OR<br>"psychological distress*"[tiab] OR<br>"ptsd"[tiab] OR<br>"ptss"[tiab] OR<br>"somatoform disorder*"[tiab] OR<br>"schizophren*"[tiab] OR<br>"stress*"[tiab]<br><br>OR<br><br>"User satisfaction"[tiab] OR<br>"acceptability"[tiab] OR<br>"feasibility"[tiab]

	"health care provider*"[tiab] OR<br>"healthcare provider*"[tiab] OR<br>"humanitarian aid*"[tiab] OR<br>"humanitarian relie*"[tiab] OR<br>"humanitarian service*"[tiab] OR<br>"humanitarian worker*"[tiab] OR<br>"medical resident*"[tiab] OR<br>"medic"[tiab] OR<br>"medics"[tiab] OR<br>"nurse*"[tiab] OR<br>"nursing"[tiab] OR<br>"paramedic*"[tiab] OR<br>"police*"[tiab] OR<br>"police m*"[tiab] OR<br>"police wom*"[tiab] OR<br>"police officer*"[tiab] OR<br>"firem*"[tiab] OR<br>"fire m*"[tiab] OR<br>"fire wom*"[tiab] OR<br>"physician*"[tiab] OR<br>"relief work*"[tiab] OR<br>"rescuer*"[tiab] OR<br>"rescue work*"[tiab])			OR<br><br>"Absenteeism"[Mesh] OR<br>"Employment"[Mesh] OR<br>"Job Satisfaction"[MeSH] OR<br>"Sick Leave"[Mesh] OR<br>"Retirement"[Mesh] OR<br>"Work Capacity Evaluation"[Mesh] OR<br>"absenteeism*"[tiab] OR<br>"back to work"[tiab] OR<br>"early retir*"[tiab] OR<br>"effectiveness"[tiab] OR<br>"employabil*"[tiab] OR<br>"employment status*"[tiab] OR<br>"job length*"[tiab] OR<br>"job retenti*"[tiab] OR<br>"job satisf*"[tiab] OR<br>"work satisf*"[tiab] OR<br>"medical certificate*"[tiab] OR<br>"presenteeism*"[tiab] OR<br>"productivit*"[tiab] OR<br>"prolonged work*"[tiab] OR<br>"prolonging work*"[tiab] OR<br>"resignati*"[tiab] OR<br>"return to work"[tiab] OR<br>"sick leav*"[tiab] OR<br>"sickness absen*"[tiab] OR<br>"sickness presen*"[tiab] OR<br>"sick listing*"[tiab] OR<br>"sustainable work*"[tiab] OR<br>"sustained work*"[tiab] OR<br>"unemploy*"[tiab] OR<br>"work absence*"[tiab] OR<br>"work abilit*"[tiab] OR<br>"work capacit*"[tiab] OR<br>"work disabilit*"[tiab] OR<br>"work engag*"[tiab] OR<br>"work function*"[tiab] OR<br>"work participati*"[tiab] OR<br>"work performan*"[tiab] OR<br>"work retention"[tiab] OR

				"turnover*"[tiab] OR<br>"turn over*"[tiab]<br><br>OR<br><br>"drop out*"[tiab] OR<br>"dropout*"[tiab]<br><br>OR<br><br>"Optimism"[Mesh] OR<br>"Personal Satisfaction"[Mesh] OR<br>"Self Concept"[Mesh:NoExp] OR<br>"Self Efficacy"[Mesh] OR<br>"Self-Control"[Mesh] OR<br>"life engag*"[tiab] OR<br>"life satisf*"[tiab] OR<br>"meaning of life"[tiab] OR<br>"purpose in life"[tiab] OR<br>"positive affect*"[tiab] OR<br>"positive emotion*"[tiab] OR<br>"resilien*"[tiab] OR<br>"self concept*"[tiab] OR<br>"self control*"[tiab] OR<br>"self efficac*"[tiab] OR<br>"self esteem*"[tiab] OR<br>"swb"[tiab] OR<br>"well being*"[tiab] OR<br>"wellbeing*"[tiab]<br><br>OR<br><br>"Quality of Life"[Mesh] OR<br>"Quality-Adjusted Life Years"[Mesh] OR<br>"disability adjusted life"[tiab] OR<br>"qaly"[tiab] OR<br>"daly"[tiab] OR<br>"functioning"[tiab] OR<br>"functional abilit*" OR<br>"functionalit*" OR<br>"hrqol*"[tiab] OR<br>"life activit*"[tiab] OR

				"life participati*"[tiab] OR<br>"life stress*"[tiab] OR<br>"qol"[tiab] OR<br>"qoli"[tiab] OR<br>"quality of life*"[tiab] OR<br>"self car*"[tiab] OR<br>"selfcar*"[tiab] OR<br>"sickness impact profile*" [tiab] OR<br>"social function*"[tiab] OR<br>"social participati*"[tiab]<br><br>OR<br><br>"Help-Seeking Behavior"[Mesh] OR<br>"helpseek*"[tiab] OR<br>"help seek*"[tiab] OR<br>"help-seek*"[tiab] OR<br>"seek help"[tiab] OR<br>"seeking help"[tiab] OR<br>"seek treatment"[tiab] OR<br>"seeking treatment"[tiab] OR<br>"treatment seeking"[tiab] OR<br>"help seeking behav*"[tiab]
**PsychINFO (Ovid)**	"Occupational Groups".MH. OR<br>"Occupational Health".MH. OR<br>"Personnel".sh. OR<br>"enterprise*".ti,ab. OR<br>"business*".ti,ab. OR<br>"employed".ti,ab. OR<br>"employee*".ti,ab. OR<br>"employer*".ti,ab. OR<br>"employment*".ti,ab. OR<br>"informal sector*".ti,ab. OR<br>"informal work*".ti,ab. OR<br>"laborer*".ti,ab. OR<br>"labourer*".ti,ab. OR<br>"occupation*".ti,ab. OR<br>"personnel*".ti,ab. OR<br>"professional".ti,ab. OR<br>"professionals".ti,ab. OR<br>"staff".ti,ab. OR	(screen*.ti,ab. OR<br>monitoring.ti,ab. OR<br>check.ti,ab. OR<br>check-up.ti,ab. OR<br>surveillance.ti,ab. OR<br>detection.ti,ab.)<br><br>AND<br><br>("employee*".ti,ab. OR<br>"laborer*".ti,ab. OR<br>"labourer*".ti,ab. OR<br>"personnel*".ti,ab. OR<br>"professionals".ti,ab. OR "staff".ti,ab. OR<br>"worker*".ti,ab. OR<br>"workforce".ti,ab. OR<br>"workplace*".ti,ab. OR	"Randomized Controlled Trial".pt. OR<br>"Randomized Controlled Trials as Topic".mh. OR<br>"Non-Randomized Controlled Trials as Topic".mh. OR<br>"Random Allocation".mh. OR<br>"RCT".ti,ab. OR<br>"randomized controlled trial".ti,ab. OR<br>"randomised controlled trial".ti,ab. OR<br>"non-randomized controlled trial".ti,ab. OR<br>"non-randomised controlled trial".ti,ab. OR<br>"non randomized controlled trial".ti,ab. OR<br>"non randomised controlled trial".ti,ab. OR<br>"random allocation".ti,ab. OR<br>"random assignment".ti,ab. OR<br>"randomized".ti,ab. OR<br>"randomised".ti,ab. OR<br>"quasi-experiment*".ti,ab. OR<br>"quasi experiment*".ti,ab. OR<br>"control group".ti,ab. OR	"Mental Disorders".mh. OR<br>"Mental Health".mh. OR<br>"Psychology, Industrial".mh. OR<br>"Stress, Psychological".mh. OR<br>"Psychological Stress".sh. OR<br>"adjustment".ti,ab. OR<br>"affective disorder*".ti,ab. OR<br>"anxiet*".ti,ab. OR<br>"bipolar*".ti,ab. OR<br>"burn out*".ti,ab. OR<br>"burnout*".ti,ab. OR<br>"CMD" .ti,ab. OR<br>"depressi*".ti,ab. OR<br>"eating disorder*".ti,ab. OR<br>"mental disorder*".ti,ab. OR<br>"mental health*".ti,ab. OR<br>"mental illness*".ti,ab. OR<br>"mood disorder*".ti,ab. OR

	"staffing*".ti,ab. OR<br>"vocation".ti,ab. OR<br>"vocations".ti,ab. OR<br>"worker*".ti,ab. OR<br>"workforce".ti,ab. OR<br>"workplace*".ti,ab. OR<br>"work place*".ti,ab. OR<br>"worksite*".ti,ab.<br><br>OR<br><br>"ambulance*".ti,ab. OR<br>"paramedic*".ti,ab. OR<br>"disaster responder*".ti,ab. OR<br>"doctor*".ti,ab. OR<br>"emergency service*".ti,ab. OR<br>"emergency responder*".ti,ab. OR<br>"emergency medical".ti,ab. OR<br>"firefighter*".ti,ab. OR<br>"fire fighter*".ti,ab. OR<br>"first responder*".ti,ab. OR<br>"general practitioner*".ti,ab. OR<br>"health care provider*".ti,ab. OR<br>"healthcare provider*".ti,ab. OR<br>"humanitarian aid*".ti,ab. OR<br>"humanitarian relie*".ti,ab. OR<br>"humanitarian service*".ti,ab. OR<br>"humanitarian worker*".ti,ab. OR<br>"medical resident*".ti,ab. OR<br>"medic".ti,ab. OR<br>"medics".ti,ab. OR<br>"nurse*".ti,ab. OR<br>"nursing".ti,ab. OR<br>"paramedic*".ti,ab. OR<br>"police*".ti,ab. OR<br>"police m*".ti,ab. OR<br>"police wom*".ti,ab. OR<br>"police officer*".ti,ab. OR<br>"firem*".ti,ab. OR<br>"fire m*".ti,ab. OR<br>"fire wom*".ti,ab. OR	"work place*".ti,ab. OR<br>"worksite*".ti,ab.)	"comparison group".ti,ab. OR<br>"wait-list control".ti,ab. OR<br>"wait list control".ti,ab. OR<br>"control condition".ti,ab.	"obsessive compulsive disorder*".ti,ab. OR<br>"ocd".ti,ab. OR<br>"panic disorder*".ti,ab. OR<br>"phobi*".ti,ab. OR<br>"post traumatic*".ti,ab. OR<br>"psychiatric diagnos*".ti,ab. OR<br>"psychiatric disease*".ti,ab. OR<br>"psychiatric disorder*".ti,ab. OR<br>"psychiatric illness*".ti,ab. OR<br>"psychological disorder*".ti,ab. OR<br>"psychos*".ti,ab. OR<br>"psychotic*".ti,ab. OR<br>"psychological distress*".ti,ab. OR<br>"ptsd".ti,ab. OR<br>"ptss".ti,ab. OR<br>"somatoform disorder*".ti,ab. OR<br>"schizophren*".ti,ab. OR<br>"stress*".ti,ab.<br><br>OR<br><br>"User satisfaction".ti,ab. OR<br>"acceptability".ti,ab. OR<br>"feasibility".ti,ab.<br><br>OR<br><br>"Absenteeism".mh. OR<br>"Employment".mh. OR<br>"Employee Efficiency".sh. OR<br>"Employee Absenteeism".sh. OR<br>"Job Satisfaction".mh. OR<br>"Sick Leave".mh. OR<br>"Employee Leave Benefit".mh. OR<br>"Retirement".mh. OR<br>"Work Capacity Evaluation".mh. OR<br>"absenteeism*".ti,ab. OR<br>"back to work".ti,ab. OR<br>"early retir*".ti,ab. OR<br>"effectiveness".ti,ab. OR<br>"employabil*".ti,ab. OR<br>"employment status*".ti,ab. OR

	"physician*".ti,ab. OR<br>"relief work*".ti,ab. OR<br>"rescuer*".ti,ab. OR<br>"rescue work*".ti,ab.			"job length*".ti,ab. OR<br>"job retenti*".ti,ab. OR<br>"job satisf*".ti,ab. OR<br>"work satisf*".ti,ab. OR<br>"medical certificate*".ti,ab. OR<br>"presenteeism*".ti,ab. OR<br>"productivit*".ti,ab. OR<br>"prolonged work*".ti,ab. OR<br>"prolonging work*".ti,ab. OR<br>"resignati*".ti,ab. OR<br>"return to work".ti,ab. OR<br>"sick leav*".ti,ab. OR<br>"sickness absen*".ti,ab. OR<br>"sickness presen*".ti,ab. OR<br>"sick listing*".ti,ab. OR<br>"sustainable work*".ti,ab. OR<br>"sustained work*".ti,ab. OR<br>"unemploy*".ti,ab. OR<br>"work absence*".ti,ab. OR<br>"work abilit*".ti,ab. OR<br>"work capacit*".ti,ab. OR<br>"work disabilit*".ti,ab. OR<br>"work engag*".ti,ab. OR<br>"work function*".ti,ab. OR<br>"work participati*".ti,ab. OR<br>"work performan*".ti,ab. OR<br>"work retention".ti,ab. OR<br>"turnover*".ti,ab. OR<br>"turn over*".ti,ab.<br><br>OR<br><br>"drop out*".ti,ab. OR<br>"dropout*".ti,ab.<br><br>OR<br><br>"Optimism".mh. OR<br>"Personal Satisfaction".mh. OR<br>"Satisfaction".sh. OR<br>"Self Concept".mh. OR<br>"Self Efficacy".mh. OR

				"Self-Control".mh. OR<br>"life engag*".ti,ab. OR<br>"life satisf*".ti,ab. OR<br>"meaning of life".ti,ab. OR<br>"purpose in life".ti,ab. OR<br>"positive affect*".ti,ab. OR<br>"positive emotion*".ti,ab. OR<br>"resilien*".ti,ab. OR<br>"self concept*".ti,ab. OR<br>"self control*".ti,ab. OR<br>"self efficac*".ti,ab. OR<br>"self esteem*".ti,ab. OR<br>"swb".ti,ab. OR<br>"well being*".ti,ab. OR<br>"wellbeing*".ti,ab.<br><br>OR<br><br>"Quality of Life".mh. OR<br>"Quality-Adjusted Life Years".mh. OR<br>"disability adjusted life".ti,ab. OR<br>"qaly".ti,ab. OR<br>"daly".ti,ab. OR<br>"functioning".ti,ab. OR<br>"functional abilit*" OR<br>"functionalit*" OR<br>"hrqol*".ti,ab. OR<br>"life activit*".ti,ab. OR<br>"life participati*".ti,ab. OR<br>"life stress*".ti,ab. OR<br>"qol".ti,ab. OR<br>"qoli".ti,ab. OR<br>"quality of life*".ti,ab. OR<br>"self car*".ti,ab. OR<br>"selfcar*".ti,ab. OR<br>"sickness impact profile*" .ti,ab. OR<br>"social function*".ti,ab. OR<br>"social participati*".ti,ab.<br><br>OR<br><br>"Help-Seeking Behavior".mh. OR

				"helpseek*".ti,ab. OR<br>"help seek*".ti,ab. OR<br>"help-seek*".ti,ab. OR<br>"seek help".ti,ab. OR<br>"seeking help".ti,ab. OR<br>"seek treatment".ti,ab. OR<br>"seeking treatment".ti,ab. OR<br>"treatment seeking".ti,ab. OR<br>"help seeking behav*".ti,ab.
**EMABSE (Ovid)**	"named groups by occupation".sh. OR<br>"Occupational Health".sh. OR<br>"enterprise*".ti,ab. OR<br>"business*".ti,ab. OR<br>"employed".ti,ab. OR<br>"employee*".ti,ab. OR<br>"employer*".ti,ab. OR<br>"employment*".ti,ab. OR<br>"informal sector*".ti,ab. OR<br>"informal work*".ti,ab. OR<br>"laborer*".ti,ab. OR<br>"labourer*".ti,ab. OR<br>"occupation*".ti,ab. OR<br>"personnel*".ti,ab. OR<br>"professional".ti,ab. OR<br>"professionals".ti,ab. OR<br>"staff".ti,ab. OR<br>"staffing*".ti,ab. OR<br>"vocation".ti,ab. OR<br>"vocations".ti,ab. OR<br>"worker*".ti,ab. OR<br>"workforce".ti,ab. OR<br>"workplace*".ti,ab. OR<br>"work place*".ti,ab. OR<br>"worksite*".ti,ab.<br><br>OR<br><br>"ambulance*".ti,ab. OR<br>"paramedic*".ti,ab. OR<br>"disaster responder*".ti,ab. OR	(screen*.ti,ab. OR<br>monitoring.ti,ab. OR<br>check.ti,ab. OR<br>check-up.ti,ab. OR<br>surveillance.ti,ab. OR<br>detection.ti,ab.)<br><br>AND<br><br>("employee*".ti,ab. OR<br>"laborer*".ti,ab. OR<br>"labourer*".ti,ab. OR<br>"personnel*".ti,ab. OR<br>"professionals".ti,ab. OR "staff".ti,ab. OR<br>"worker*".ti,ab. OR<br>"workforce".ti,ab. OR<br>"workplace*".ti,ab. OR<br>"work place*".ti,ab. OR<br>"worksite*".ti,ab.)	"Randomized Controlled Trial".pt. OR<br>"Randomized Controlled Trials as Topic".sh. OR<br>"Non-Randomized Controlled Trials as Topic".sh. OR<br>"Random Allocation".sh. OR<br>"RCT".ti,ab. OR<br>"randomized controlled trial".ti,ab. OR<br>"randomised controlled trial".ti,ab. OR<br>"non-randomized controlled trial".ti,ab. OR<br>"non-randomised controlled trial".ti,ab. OR<br>"non randomized controlled trial".ti,ab. OR<br>"non randomised controlled trial".ti,ab. OR<br>"random allocation".ti,ab. OR<br>"random assignment".ti,ab. OR<br>"randomized".ti,ab. OR<br>"randomised".ti,ab. OR<br>"quasi-experiment*".ti,ab. OR<br>"quasi experiment*".ti,ab. OR<br>"control group".ti,ab. OR<br>"comparison group".ti,ab. OR<br>"wait-list control".ti,ab. OR<br>"wait list control".ti,ab. OR<br>"control condition".ti,ab.	"Mental Disease".sh. OR<br>"Mental Health".sh. OR<br>"mental stress".sh. OR<br>"adjustment".ti,ab. OR<br>"affective disorder*".ti,ab. OR<br>"anxiet*".ti,ab. OR<br>"bipolar*".ti,ab. OR<br>"burn out*".ti,ab. OR<br>"burnout*".ti,ab. OR<br>"CMD" .ti,ab. OR<br>"depressi*".ti,ab. OR<br>"eating disorder*".ti,ab. OR<br>"mental disorder*".ti,ab. OR<br>"mental health*".ti,ab. OR<br>"mental illness*".ti,ab. OR<br>"mood disorder*".ti,ab. OR<br>"obsessive compulsive disorder*".ti,ab. OR<br>"ocd".ti,ab. OR<br>"panic disorder*".ti,ab. OR<br>"phobi*".ti,ab. OR<br>"post traumatic*".ti,ab. OR<br>"psychiatric diagnos*".ti,ab. OR<br>"psychiatric disease*".ti,ab. OR<br>"psychiatric disorder*".ti,ab. OR<br>"psychiatric illness*".ti,ab. OR<br>"psychological disorder*".ti,ab. OR<br>"psychos*".ti,ab. OR<br>"psychotic*".ti,ab. OR<br>"psychological distress*".ti,ab. OR<br>"ptsd".ti,ab. OR<br>"ptss".ti,ab. OR<br>"somatoform disorder*".ti,ab. OR

	"doctor*".ti,ab. OR<br>"emergency service*".ti,ab. OR<br>"emergency responder*".ti,ab. OR<br>"emergency medical".ti,ab. OR<br>"firefighter*".ti,ab. OR<br>"fire fighter*".ti,ab. OR<br>"first responder*".ti,ab. OR<br>"general practitioner*".ti,ab. OR<br>"health care provider*".ti,ab. OR<br>"healthcare provider*".ti,ab. OR<br>"humanitarian aid*".ti,ab. OR<br>"humanitarian relie*".ti,ab. OR<br>"humanitarian service*".ti,ab. OR<br>"humanitarian worker*".ti,ab. OR<br>"medical resident*".ti,ab. OR<br>"medic".ti,ab. OR<br>"medics".ti,ab. OR<br>"nurse*".ti,ab. OR<br>"nursing".ti,ab. OR<br>"paramedic*".ti,ab. OR<br>"police*".ti,ab. OR<br>"police m*".ti,ab. OR<br>"police wom*".ti,ab. OR<br>"police officer*".ti,ab. OR<br>"firem*".ti,ab. OR<br>"fire m*".ti,ab. OR<br>"fire wom*".ti,ab. OR<br>"physician*".ti,ab. OR<br>"relief work*".ti,ab. OR<br>"rescuer*".ti,ab. OR<br>"rescue work*".ti,ab.			"schizophren*".ti,ab. OR<br>"stress*".ti,ab.<br><br>OR<br><br>"User satisfaction".ti,ab. OR<br>"acceptability".ti,ab. OR<br>"feasibility".ti,ab.<br><br>OR<br><br>"Absenteeism".sh. OR<br>"Employment".sh. OR<br>"Job Satisfaction".sh. OR<br>"Medical Leave".sh. OR<br>"Retirement".sh. OR<br>"Work Capacity".sh. OR<br>"absenteeism*".ti,ab. OR<br>"back to work".ti,ab. OR<br>"early retir*".ti,ab. OR<br>"effectiveness".ti,ab. OR<br>"employabil*".ti,ab. OR<br>"employment status*".ti,ab. OR<br>"job length*".ti,ab. OR<br>"job retenti*".ti,ab. OR<br>"job satisf*".ti,ab. OR<br>"work satisf*".ti,ab. OR<br>"medical certificate*".ti,ab. OR<br>"presenteeism*".ti,ab. OR<br>"productivit*".ti,ab. OR<br>"prolonged work*".ti,ab. OR<br>"prolonging work*".ti,ab. OR<br>"resignati*".ti,ab. OR<br>"return to work".ti,ab. OR<br>"sick leav*".ti,ab. OR<br>"sickness absen*".ti,ab. OR<br>"sickness presen*".ti,ab. OR<br>"sick listing*".ti,ab. OR<br>"sustainable work*".ti,ab. OR<br>"sustained work*".ti,ab. OR<br>"unemploy*".ti,ab. OR<br>"work absence*".ti,ab. OR

				"work abilit*".ti,ab. OR<br>"work capacit*".ti,ab. OR<br>"work disabilit*".ti,ab. OR<br>"work engag*".ti,ab. OR<br>"work function*".ti,ab. OR<br>"work participati*".ti,ab. OR<br>"work performan*".ti,ab. OR<br>"work retention".ti,ab. OR<br>"turnover*".ti,ab. OR<br>"turn over*".ti,ab.<br><br>OR<br><br>"drop out*".ti,ab. OR<br>"dropout*".ti,ab.<br><br>OR<br><br>"Optimism".sh. OR<br>"Satisfaction".sh. OR<br>"Self Concept".sh. OR<br>"Self Control".sh. OR<br>"life engag*".ti,ab. OR<br>"life satisf*".ti,ab. OR<br>"meaning of life".ti,ab. OR<br>"purpose in life".ti,ab. OR<br>"positive affect*".ti,ab. OR<br>"positive emotion*".ti,ab. OR<br>"resilien*".ti,ab. OR<br>"self concept*".ti,ab. OR<br>"self control*".ti,ab. OR<br>"self efficac*".ti,ab. OR<br>"self esteem*".ti,ab. OR<br>"swb".ti,ab. OR<br>"well being*".ti,ab. OR<br>"wellbeing*".ti,ab.<br><br>OR<br><br>"Quality of Life".sh. OR<br>"Quality-Adjusted Life Year".sh. OR<br>"disability adjusted life".ti,ab. OR

				"qaly".ti,ab. OR<br>"daly".ti,ab. OR<br>"functioning".ti,ab. OR<br>"functional abilit*" OR<br>"functionalit*" OR<br>"hrqol*".ti,ab. OR<br>"life activit*".ti,ab. OR<br>"life participati*".ti,ab. OR<br>"life stress*".ti,ab. OR<br>"qol".ti,ab. OR<br>"qoli".ti,ab. OR<br>"quality of life*".ti,ab. OR<br>"self car*".ti,ab. OR<br>"selfcar*".ti,ab. OR<br>"sickness impact profile*" .ti,ab. OR<br>"social function*".ti,ab. OR<br>"social participati*".ti,ab.<br><br>OR<br><br>"Help-Seeking Behavior".sh. OR<br>"helpseek*".ti,ab. OR<br>"help seek*".ti,ab. OR<br>"help-seek*".ti,ab. OR<br>"seek help".ti,ab. OR<br>"seeking help".ti,ab. OR<br>"seek treatment".ti,ab. OR<br>"seeking treatment".ti,ab. OR<br>"treatment seeking".ti,ab. OR<br>"help seeking behav*".ti,ab.
**CENTRAL**	[mh "Occupational Groups"] OR<br>[mh "Occupational Health"] OR<br>"enterprise*" OR<br>"business*" OR<br>"employed" OR<br>"employee*" OR<br>"employer*" OR<br>"employment*" OR<br>"informal sector*" OR<br>"informal work*" OR<br>"laborer*" OR	(screen* OR<br>monitoring OR<br>check OR<br>check-up OR<br>surveillance OR<br>detection)<br><br>AND<br><br>("employee*" OR<br>"laborer*" OR	("Randomized Controlled Trial"):pt OR<br>[mh "Randomized Controlled Trials as Topic"] OR<br>[mh "Non-Randomized Controlled Trials as Topic"] OR<br>[mh "Random Allocation"] OR<br>"RCT" OR<br>"randomized controlled trial" OR<br>"randomised controlled trial" OR<br>"non-randomized controlled trial" OR<br>"non-randomised controlled trial" OR<br>"non randomized controlled trial" OR<br>"non randomised controlled trial" OR	[mh "Mental Disorders"] OR<br>[mh "Mental Health"] OR<br>[mh "Psychology, Industrial"] OR<br>[mh "Stress, Psychological"] OR<br>"adjustment" OR<br>"affective disorder*" OR<br>"anxiet*" OR<br>"bipolar*" OR<br>"burn out*" OR<br>"burnout*" OR<br>"CMD" OR

	"labourer*" OR<br>"occupation*" OR<br>"personnel*" OR<br>"professional" OR<br>"professionals" OR<br>"staff" OR<br>"staffing*" OR<br>"vocation" OR<br>"vocations" OR<br>"worker*" OR<br>"workforce" OR<br>"workplace*" OR<br>"work place*" OR<br>"worksite*"<br><br>OR<br><br>"ambulance*" OR<br>"paramedic*" OR<br>"disaster responder*" OR<br>"doctor*" OR<br>"emergency service*" OR<br>"emergency responder*" OR<br>"emergency medical" OR<br>"firefighter*" OR<br>"fire fighter*" OR<br>"first responder*" OR<br>"general practitioner*" OR<br>"health care provider*" OR<br>"healthcare provider*" OR<br>"humanitarian aid*" OR<br>"humanitarian relie*" OR<br>"humanitarian service*" OR<br>"humanitarian worker*" OR<br>"medical resident*" OR<br>"medic" OR<br>"medics" OR<br>"nurse*" OR<br>"nursing" OR<br>"paramedic*" OR<br>"police*" OR<br>"police m*" OR	"labourer*" OR<br>"personnel*" OR<br>"professionals" OR<br>"staff" OR<br>"worker*" OR<br>"workforce" OR<br>"workplace*" OR<br>"work place*" OR<br>"worksite*")	"random allocation" OR<br>"random assignment" OR<br>"randomized" OR<br>"randomised" OR<br>"quasi-experiment*" OR<br>"quasi experiment*" OR<br>"control group" OR<br>"comparison group" OR<br>"wait-list control" OR<br>"wait list control" OR<br>"control condition"	"depressi*" OR<br>"eating disorder*" OR<br>"mental disorder*" OR<br>"mental health*" OR<br>"mental illness*" OR<br>"mood disorder*" OR<br>"obsessive compulsive disorder*" OR<br>"ocd" OR<br>"panic disorder*" OR<br>"phobi*" OR<br>"post traumatic*" OR<br>"psychiatric diagnos*" OR<br>"psychiatric disease*" OR<br>"psychiatric disorder*" OR<br>"psychiatric illness*" OR<br>"psychological disorder*" OR<br>"psychos*" OR<br>"psychotic*" OR<br>"psychological distress*" OR<br>"ptsd" OR<br>"ptss" OR<br>"somatoform disorder*" OR<br>"schizophren*" OR<br>"stress*"<br><br>OR<br><br>"User satisfaction" OR<br>"acceptability" OR<br>"feasibility"<br><br>OR<br><br>[mh "Absenteeism"] OR<br>[mh "Employment"] OR<br>[mh "Job Satisfaction"] OR<br>[mh "Sick Leave"] OR<br>[mh "Retirement"] OR<br>[mh "Work Capacity Evaluation"] OR<br>"absenteeism*" OR<br>"back to work" OR<br>"early retir*" OR

	"police wom*" OR<br>"police officer*" OR<br>"firem*" OR<br>"fire m*" OR<br>"fire wom*" OR<br>"physician*" OR<br>"relief work*" OR<br>"rescuer*" OR<br>"rescue work*"			"effectiveness" OR<br>"employabil*" OR<br>"employment status*" OR<br>"job length*" OR<br>"job retenti*" OR<br>"job satisf*" OR<br>"work satisf*" OR<br>"medical certificate*" OR<br>"presenteeism*" OR<br>"productivit*" OR<br>"prolonged work*" OR<br>"prolonging work*" OR<br>"resignati*" OR<br>"return to work" OR<br>"sick leav*" OR<br>"sickness absen*" OR<br>"sickness presen*" OR<br>"sick listing*" OR<br>"sustainable work*" OR<br>"sustained work*" OR<br>"unemploy*" OR<br>"work absence*" OR<br>"work abilit*" OR<br>"work capacit*" OR<br>"work disabilit*" OR<br>"work engag*" OR<br>"work function*" OR<br>"work participati*" OR<br>"work performan*" OR<br>"work retention" OR<br>"turnover*" OR<br>"turn over*"<br><br>OR<br><br>"drop out*" OR<br>"dropout*"<br><br>OR<br><br>[mh "Optimism"] OR<br>[mh "Personal Satisfaction"] OR

				[mh "Self Concept"] OR<br>[mh "Self Efficacy"] OR<br>[mh "Self-Control"] OR<br>"life engag*" OR<br>"life satisf*" OR<br>"meaning of life" OR<br>"purpose in life" OR<br>"positive affect*" OR<br>"positive emotion*" OR<br>"resilien*" OR<br>"self concept*" OR<br>"self control*" OR<br>"self efficac*" OR<br>"self esteem*" OR<br>"swb" OR<br>"well being*" OR<br>"wellbeing*"<br><br>OR<br><br>[mh "Quality of Life"] OR<br>[mh "Quality-Adjusted Life Years"] OR<br>"disability adjusted life" OR<br>"qaly" OR<br>"daly" OR<br>"functioning" OR<br>"functional abilit*" OR<br>"functionalit*" OR<br>"hrqol*" OR<br>"life activit*" OR<br>"life participati*" OR<br>"life stress*" OR<br>"qol" OR<br>"qoli" OR<br>"quality of life*" OR<br>"self car*" OR<br>"selfcar*" OR<br>"sickness impact profile*" OR<br>"social function*" OR<br>"social participati*"<br><br>OR

				[mh "Help-Seeking Behavior"] OR<br>"helpseek*" OR<br>"help seek*" OR<br>"help-seek*" OR<br>"seek help" OR<br>"seeking help" OR<br>"seek treatment" OR<br>"seeking treatment" OR<br>"treatment seeking" OR<br>"help seeking behav*"
**Global Index Medicus**	mh:(Occupational Groups) OR<br>mh:(Occupational Health) OR<br>"enterprise*" OR<br>"business*" OR<br>"employed" OR<br>"employee*" OR<br>"employer*" OR<br>"employment*" OR<br>"informal sector*" OR<br>"informal work*" OR<br>"laborer*" OR<br>"labourer*" OR<br>"occupation*" OR<br>"personnel*" OR<br>"professional" OR<br>"professionals" OR<br>"staff" OR<br>"staffing*" OR<br>"vocation" OR<br>"vocations" OR<br>"worker*" OR<br>"workforce" OR<br>"workplace*" OR<br>"work place*" OR<br>"worksite*"<br><br>OR<br><br>"ambulance*" OR<br>"paramedic*" OR<br>"disaster responder*" OR	(screen* OR<br>monitoring OR<br>check OR<br>check-up OR<br>surveillance OR<br>detection)<br><br>AND<br><br>("employee*" OR<br>"laborer*" OR<br>"labourer*" OR<br>"personnel*" OR<br>"professionals" OR<br>"staff" OR<br>"worker*" OR<br>"workforce" OR<br>"workplace*" OR<br>"work place*" OR<br>"worksite*")	mh:(Randomized Controlled Trials as Topic) OR<br>mh:(Non-Randomized Controlled Trials as Topic) OR<br>mh:(Random Allocation) OR<br>"RCT" OR<br>"randomized controlled trial" OR<br>"randomised controlled trial" OR<br>"non-randomized controlled trial" OR<br>"non-randomised controlled trial" OR<br>"non randomized controlled trial" OR<br>"non randomised controlled trial" OR<br>"random allocation" OR<br>"random assignment" OR<br>"randomized" OR<br>"randomised" OR<br>"quasi-experiment*" OR<br>"quasi experiment*" OR<br>"control group" OR<br>"comparison group" OR<br>"wait-list control" OR<br>"wait list control" OR<br>"control condition"	mh:(Mental Disease) OR<br>mh:(Mental Health) OR<br>mh:(mental stress) OR<br>"adjustment" OR<br>"affective disorder*" OR<br>"anxiet*" OR<br>"bipolar*" OR<br>"burn out*" OR<br>"burnout*" OR<br>"CMD" OR<br>"depressi*" OR<br>"eating disorder*" OR<br>"mental disorder*" OR<br>"mental health*" OR<br>"mental illness*" OR<br>"mood disorder*" OR<br>"obsessive compulsive disorder*" OR<br>"ocd" OR<br>"panic disorder*" OR<br>"phobi*" OR<br>"post traumatic*" OR<br>"psychiatric diagnos*" OR<br>"psychiatric disease*" OR<br>"psychiatric disorder*" OR<br>"psychiatric illness*" OR<br>"psychological disorder*" OR<br>"psychos*" OR<br>"psychotic*" OR<br>"psychological distress*" OR<br>"ptsd" OR<br>"ptss" OR

	"doctor*" OR<br>"emergency service*" OR<br>"emergency responder*" OR<br>"emergency medical" OR<br>"firefighter*" OR<br>"fire fighter*" OR<br>"first responder*" OR<br>"general practitioner*" OR<br>"health care provider*" OR<br>"healthcare provider*" OR<br>"humanitarian aid*" OR<br>"humanitarian relie*" OR<br>"humanitarian service*" OR<br>"humanitarian worker*" OR<br>"medical resident*" OR<br>"medic" OR<br>"medics" OR<br>"nurse*" OR<br>"nursing" OR<br>"paramedic*" OR<br>"police*" OR<br>"police m*" OR<br>"police wom*" OR<br>"police officer*" OR<br>"firem*" OR<br>"fire m*" OR<br>"fire wom*" OR<br>"physician*" OR<br>"relief work*" OR<br>"rescuer*" OR<br>"rescue work*"			"somatoform disorder*" OR<br>"schizophren*" OR<br>"stress*"<br><br>OR<br><br>"User satisfaction" OR<br>"acceptability" OR<br>"feasibility"<br><br>OR<br><br>mh:(Absenteeism) OR<br>mh:(Employment) OR<br>mh:(Job Satisfaction) OR<br>mh:(Medical Leave) OR<br>mh:(Retirement) OR<br>mh:(Work Capacity) OR<br>"absenteeism*" OR<br>"back to work" OR<br>"early retir*" OR<br>"effectiveness" OR<br>"employabil*" OR<br>"employment status*" OR<br>"job length*" OR<br>"job retenti*" OR<br>"job satisf*" OR<br>"work satisf*" OR<br>"medical certificate*" OR<br>"presenteeism*" OR<br>"productivit*" OR<br>"prolonged work*" OR<br>"prolonging work*" OR<br>"resignati*" OR<br>"return to work" OR<br>"sick leav*" OR<br>"sickness absen*" OR<br>"sickness presen*" OR<br>"sick listing*" OR<br>"sustainable work*" OR<br>"sustained work*" OR<br>"unemploy*" OR

				"work absence*" OR<br>"work abilit*" OR<br>"work capacit*" OR<br>"work disabilit*" OR<br>"work engag*" OR<br>"work function*" OR<br>"work participati*" OR<br>"work performan*" OR<br>"work retention" OR<br>"turnover*" OR<br>"turn over*"<br><br>OR<br><br>"drop out*" OR<br>"dropout*"<br><br>OR<br><br>mh:(Optimism) OR<br>mh:(Satisfaction) OR<br>mh:(Self Concept) OR<br>mh:(Self Control) OR<br>"life engag*" OR<br>"life satisf*" OR<br>"meaning of life" OR<br>"purpose in life" OR<br>"positive affect*" OR<br>"positive emotion*" OR<br>"resilien*" OR<br>"self concept*" OR<br>"self control*" OR<br>"self efficac*" OR<br>"self esteem*" OR<br>"swb" OR<br>"well being*" OR<br>"wellbeing*"<br><br>OR<br><br>mh:(Quality of Life) OR<br>mh:(Quality-Adjusted Life Year) OR

				"disability adjusted life" OR<br>"qaly" OR<br>"daly" OR<br>"functioning" OR<br>"functional abilit*" OR<br>"functionalit*" OR<br>"hrqol*" OR<br>"life activit*" OR<br>"life participati*" OR<br>"life stress*" OR<br>"qol" OR<br>"qoli" OR<br>"quality of life*" OR<br>"self car*" OR<br>"selfcar*" OR<br>"sickness impact profile*" OR<br>"social function*" OR<br>"social participati*"<br><br>OR<br><br>mh:(Help-Seeking Behavior) OR<br>"helpseek*" OR<br>"help seek*" OR<br>"help-seek*" OR<br>"seek help" OR<br>"seeking help" OR<br>"seek treatment" OR<br>"seeking treatment" OR<br>"treatment seeking" OR<br>"help seeking behav*"
**Global Health**	"Occupational Health".sh. OR<br>"Personnel".sh. OR<br>"enterprise*".ti,ab. OR<br>"business*".ti,ab. OR<br>"employed".ti,ab. OR<br>"employee*".ti,ab. OR<br>"employer*".ti,ab. OR<br>"employment*".ti,ab. OR<br>"informal sector*".ti,ab. OR<br>"informal work*".ti,ab. OR<br>"laborer*".ti,ab. OR	(screen*.ti,ab. OR<br>monitoring.ti,ab. OR<br>check.ti,ab. OR<br>check-up.ti,ab. OR<br>surveillance.ti,ab. OR<br>detection.ti,ab.)<br><br>AND<br><br>("employee*".ti,ab. OR<br>"laborer*".ti,ab. OR	"Randomized Controlled Trial".pt. OR<br>"Randomized Controlled Trials as Topic".sh. OR<br>"RCT".ti,ab. OR<br>"randomized controlled trial".ti,ab. OR<br>"randomised controlled trial".ti,ab. OR<br>"non-randomized controlled trial".ti,ab. OR<br>"non-randomised controlled trial".ti,ab. OR<br>"non randomized controlled trial".ti,ab. OR<br>"non randomised controlled trial".ti,ab. OR<br>"random allocation".ti,ab. OR<br>"random assignment".ti,ab. OR	"Mental Disorders".sh. OR<br>"Mental Health".sh. OR<br>"Mental Stress".sh. OR<br>"adjustment".ti,ab. OR<br>"affective disorder*".ti,ab. OR<br>"anxiet*".ti,ab. OR<br>"bipolar*".ti,ab. OR<br>"burn out*".ti,ab. OR<br>"burnout*".ti,ab. OR<br>"CMD" .ti,ab. OR<br>"depressi*".ti,ab. OR

	"labourer*".ti,ab. OR<br>"occupation*".ti,ab. OR<br>"personnel*".ti,ab. OR<br>"professional".ti,ab. OR<br>"professionals".ti,ab. OR<br>"staff".ti,ab. OR<br>"staffing*".ti,ab. OR<br>"vocation".ti,ab. OR<br>"vocations".ti,ab. OR<br>"worker*".ti,ab. OR<br>"workforce".ti,ab. OR<br>"workplace*".ti,ab. OR<br>"work place*".ti,ab. OR<br>"worksite*".ti,ab.<br><br>OR<br><br>"ambulance*".ti,ab. OR<br>"paramedic*".ti,ab. OR<br>"disaster responder*".ti,ab. OR<br>"doctor*".ti,ab. OR<br>"emergency service*".ti,ab. OR<br>"emergency responder*".ti,ab. OR<br>"emergency medical".ti,ab. OR<br>"firefighter*".ti,ab. OR<br>"fire fighter*".ti,ab. OR<br>"first responder*".ti,ab. OR<br>"general practitioner*".ti,ab. OR<br>"health care provider*".ti,ab. OR<br>"healthcare provider*".ti,ab. OR<br>"humanitarian aid*".ti,ab. OR<br>"humanitarian relie*".ti,ab. OR<br>"humanitarian service*".ti,ab. OR<br>"humanitarian worker*".ti,ab. OR<br>"medical resident*".ti,ab. OR<br>"medic".ti,ab. OR<br>"medics".ti,ab. OR<br>"nurse*".ti,ab. OR<br>"nursing".ti,ab. OR<br>"paramedic*".ti,ab. OR<br>"police*".ti,ab. OR	"labourer*".ti,ab. OR<br>"personnel*".ti,ab. OR<br>"professionals".ti,ab. OR "staff".ti,ab. OR<br>"worker*".ti,ab. OR<br>"workforce".ti,ab. OR<br>"workplace*".ti,ab. OR<br>"work place*".ti,ab. OR<br>"worksite*".ti,ab.)	"randomized".ti,ab. OR<br>"randomised".ti,ab. OR<br>"quasi-experiment*".ti,ab. OR<br>"quasi experiment*".ti,ab. OR<br>"control group".ti,ab. OR<br>"comparison group".ti,ab. OR<br>"wait-list control".ti,ab. OR<br>"wait list control".ti,ab. OR<br>"control condition".ti,ab.	"eating disorder*".ti,ab. OR<br>"mental disorder*".ti,ab. OR<br>"mental health*".ti,ab. OR<br>"mental illness*".ti,ab. OR<br>"mood disorder*".ti,ab. OR<br>"obsessive compulsive disorder*".ti,ab. OR<br>"ocd".ti,ab. OR<br>"panic disorder*".ti,ab. OR<br>"phobi*".ti,ab. OR<br>"post traumatic*".ti,ab. OR<br>"psychiatric diagnos*".ti,ab. OR<br>"psychiatric disease*".ti,ab. OR<br>"psychiatric disorder*".ti,ab. OR<br>"psychiatric illness*".ti,ab. OR<br>"psychological disorder*".ti,ab. OR<br>"psychos*".ti,ab. OR<br>"psychotic*".ti,ab. OR<br>"psychological distress*".ti,ab. OR<br>"ptsd".ti,ab. OR<br>"ptss".ti,ab. OR<br>"somatoform disorder*".ti,ab. OR<br>"schizophren*".ti,ab. OR<br>"stress*".ti,ab.<br><br>OR<br><br>"User satisfaction".ti,ab. OR<br>"acceptability".ti,ab. OR<br>"feasibility".ti,ab.<br><br>OR<br><br>"Employment".sh. OR<br>"Employee Efficiency".sh. OR<br>"Employee Absenteeism".sh. OR<br>"Work Satisfaction".sh. OR<br>"Retirement".sh. OR<br>"absenteeism*".ti,ab. OR<br>"back to work".ti,ab. OR<br>"early retir*".ti,ab. OR<br>"effectiveness".ti,ab. OR<br>"employabil*".ti,ab. OR

	"police m*".ti,ab. OR<br>"police wom*".ti,ab. OR<br>"police officer*".ti,ab. OR<br>"firem*".ti,ab. OR<br>"fire m*".ti,ab. OR<br>"fire wom*".ti,ab. OR<br>"physician*".ti,ab. OR<br>"relief work*".ti,ab. OR<br>"rescuer*".ti,ab. OR<br>"rescue work*".ti,ab.			"employment status*".ti,ab. OR<br>"job length*".ti,ab. OR<br>"job retenti*".ti,ab. OR<br>"job satisf*".ti,ab. OR<br>"work satisf*".ti,ab. OR<br>"medical certificate*".ti,ab. OR<br>"presenteeism*".ti,ab. OR<br>"productivit*".ti,ab. OR<br>"prolonged work*".ti,ab. OR<br>"prolonging work*".ti,ab. OR<br>"resignati*".ti,ab. OR<br>"return to work".ti,ab. OR<br>"sick leav*".ti,ab. OR<br>"sickness absen*".ti,ab. OR<br>"sickness presen*".ti,ab. OR<br>"sick listing*".ti,ab. OR<br>"sustainable work*".ti,ab. OR<br>"sustained work*".ti,ab. OR<br>"unemploy*".ti,ab. OR<br>"work absence*".ti,ab. OR<br>"work abilit*".ti,ab. OR<br>"work capacit*".ti,ab. OR<br>"work disabilit*".ti,ab. OR<br>"work engag*".ti,ab. OR<br>"work function*".ti,ab. OR<br>"work participati*".ti,ab. OR<br>"work performan*".ti,ab. OR<br>"work retention".ti,ab. OR<br>"turnover*".ti,ab. OR<br>"turn over*".ti,ab.<br><br>OR<br><br>"drop out*".ti,ab. OR<br>"dropout*".ti,ab.<br><br>OR<br><br>"life engag*".ti,ab. OR<br>"life satisf*".ti,ab. OR<br>"meaning of life".ti,ab. OR<br>"purpose in life".ti,ab. OR

				"positive affect*".ti,ab. OR<br>"positive emotion*".ti,ab. OR<br>"resilien*".ti,ab. OR<br>"self concept*".ti,ab. OR<br>"self control*".ti,ab. OR<br>"self efficac*".ti,ab. OR<br>"self esteem*".ti,ab. OR<br>"swb".ti,ab. OR<br>"well being*".ti,ab. OR<br>"wellbeing*".ti,ab.<br><br>OR<br><br>"Quality of Life".sh. OR<br>"disability adjusted life".ti,ab. OR<br>"qaly".ti,ab. OR<br>"daly".ti,ab. OR<br>"functioning".ti,ab. OR<br>"functional abilit*" OR<br>"functionalit*" OR<br>"hrqol*".ti,ab. OR<br>"life activit*".ti,ab. OR<br>"life participati*".ti,ab. OR<br>"life stress*".ti,ab. OR<br>"qol".ti,ab. OR<br>"qoli".ti,ab. OR<br>"quality of life*".ti,ab. OR<br>"self car*".ti,ab. OR<br>"selfcar*".ti,ab. OR<br>"sickness impact profile*" .ti,ab. OR<br>"social function*".ti,ab. OR<br>"social participati*".ti,ab.<br><br>OR<br><br>"helpseek*".ti,ab. OR<br>"help seek*".ti,ab. OR<br>"help-seek*".ti,ab. OR<br>"seek help".ti,ab. OR<br>"seeking help".ti,ab. OR<br>"seek treatment".ti,ab. OR<br>"seeking treatment".ti,ab. OR

				"treatment seeking".ti,ab. OR "help seeking behav*".ti,ab.
**SciELO**	("enterprise*" OR "business*" OR "employed" OR "employee*" OR "employer*" OR "employment*" OR "informal sector*" OR "informal work*" OR "laborer*" OR "labourer*" OR "occupation*" OR "personnel*" OR "professional" OR "professionals" OR "staff" OR "staffing*" OR "vocation" OR "vocations" OR "worker*" OR "workforce" OR "workplace*" OR "work place*" OR "worksite*" OR "ambulance*" OR "paramedic*" OR "disaster responder*" OR "doctor*" OR "emergency service*" OR "emergency responder*" OR "emergency medical" OR "firefighter*" OR "fire fighter*" OR "first responder*" OR "general practitioner*" OR "health care provider*" OR "healthcare provider*" OR "humanitarian aid*" OR "humanitarian relie*" OR "humanitarian service*" OR "humanitarian worker*" OR "medical resident*" OR "medic" OR "medics" OR "nurse*" OR "nursing" OR "paramedic*" OR "police*" OR "police m*" OR "police wom*" OR "police officer*" OR "firem*" OR "fire m*" OR "fire wom*" OR "physician*" OR "relief work*" OR "rescuer*" OR "rescue work*")	(screen* OR monitoring OR check OR check-up OR surveillance OR detection)	("RCT" OR "randomized controlled trial" OR "randomised controlled trial" OR "non-randomized controlled trial" OR "non-randomised controlled trial" OR "non randomized controlled trial" OR "non randomised controlled trial" OR "random allocation" OR "random assignment" OR "randomized" OR "randomised" OR "quasi-experiment*" OR "quasi experiment*" OR "control group" OR "comparison group" OR "wait-list control" OR "waitlist control" OR "control condition")	("adjustment" OR "affective disorder*" OR "anxiet*" OR "bipolar*" OR "burn out*" OR "burnout*" OR "CMD" OR "depressi*" OR "eating disorder*" OR "mental disorder*" OR "mental health*" OR "mental illness*" OR "mood disorder*" OR "obsessive compulsive disorder*" OR "ocd" OR "panic disorder*" OR "phobi*" OR "post traumatic*" OR "psychiatric diagnos*" OR "psychiatric disease*" OR "psychiatric disorder*" OR "psychiatric illness*" OR "psychological disorder*" OR "psychos*" OR "psychotic*" OR "psychological distress*" OR "ptsd" OR "ptss" OR "somatoform disorder*" OR "schizophren*" OR "stress*" OR "User satisfaction" OR "acceptability" OR "feasibility" OR "absenteeism*" OR "back to work" OR "early retir*" OR "effectiveness" OR "employabil*" OR "employment status*" OR "job length*" OR "job retenti*" OR "job satisf*" OR "work satisf*" OR "medical certificate*" OR "presenteeism*" OR "productivit*" OR "prolonged work*" OR "prolonging work*" OR "resignati*" OR "return to work" OR "sick leav*" OR "sickness absen*" OR "sickness presen*" OR "sick listing*" OR "sustainable work*" OR "sustained work*" OR "unemploy*" OR "work absence*" OR "work abilit*" OR "work capacit*" OR "work disabilit*" OR "work engag*" OR "work function*" OR "work participati*" OR "work performan*" OR "work retention" OR "turnover*" OR "turn over*" OR "drop out*" OR "dropout*" OR "life engag*" OR "life satisf*" OR "meaning of life" OR "purpose in life" OR "positive affect*" OR "positive emotion*" OR "resilien*" OR "self concept*" OR "self control*" OR "self efficac*" OR "self esteem*" OR "swb" OR "well being*" OR "wellbeing*" OR "disability adjusted life" OR "qaly" OR "daly" OR "functioning" OR "functional abilit*" OR "functionalit*" OR "hrqol*" OR "life activit*" OR "life participati*" OR "life stress*" OR "qol" OR "qoli" OR "quality of life*" OR "self car*" OR "selfcar*" OR "sickness impact profile*" OR "social function*" OR

				"social participati*" OR "helpseek*" OR "help seek*" OR "help-seek*" OR "seek help" OR "seeking help" OR "seek treatment" OR "seeking treatment" OR "treatment seeking" OR "help seeking behav*")
