# Supplementary material for: Workplace mental health screening: a systematic review and meta-analysis
Source: Occup Environ Med. 2023 Jun 15;80(8):469–84. doi: 10.1136/oemed-2022-108608 (PMC10423530; doi:10.1136/oemed-2022-108608)
Supplement: Supplementary data [file oemed-2022-108608supp002.pdf]

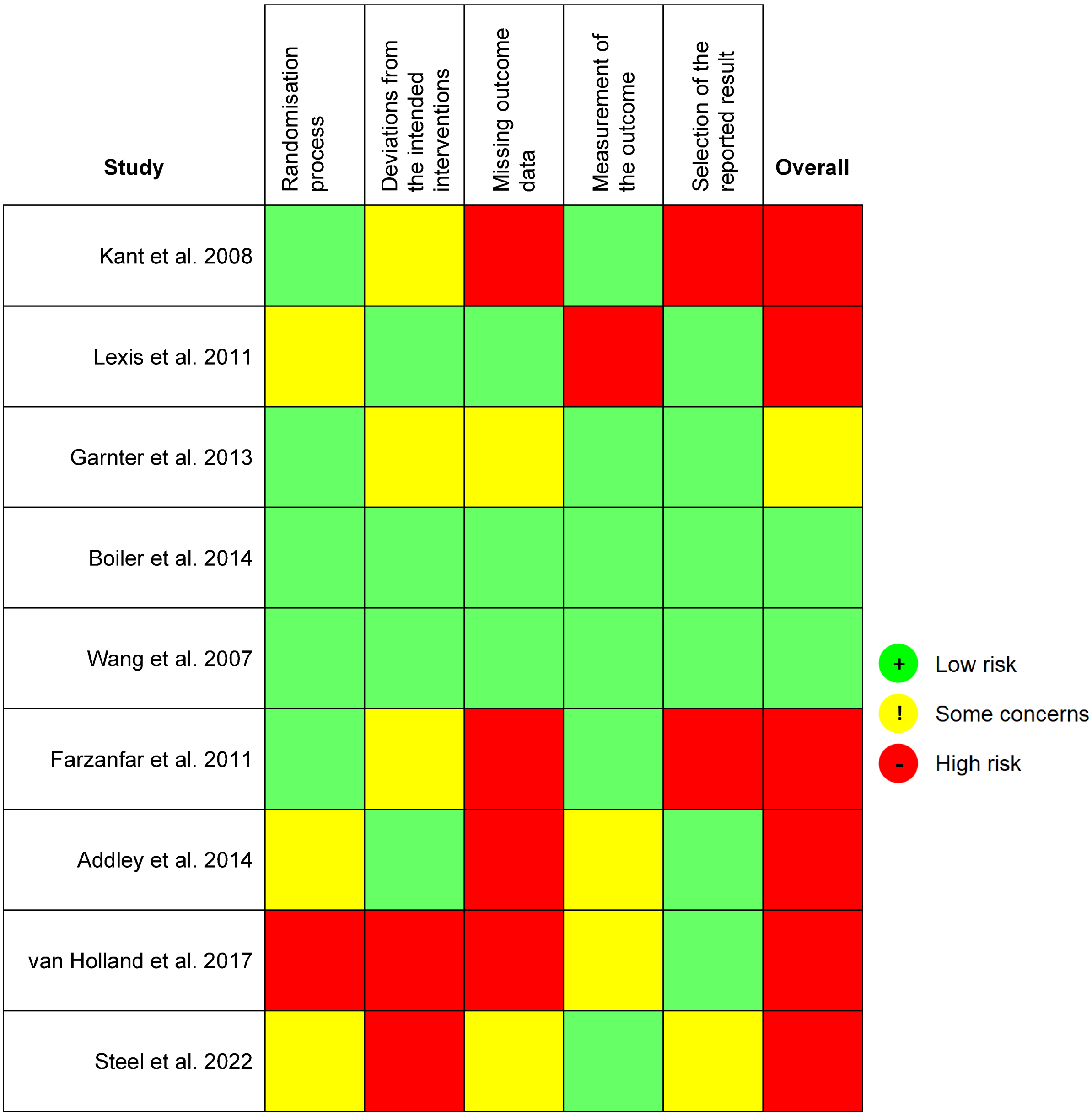

Figure 1. Summary risk of bias judgements in each domain, and overall, for each study. Note. Farzanfar et al. (2012) and Addley et al. (2014) assess risk of bias pertaining to per-protocol effect. All other studies focus on intention to treat data.
