# Supplementary material for: Workplace mental health screening: a systematic review and meta-analysis
Source: Occup Environ Med. 2023 Jun 15;80(8):469–84. doi: 10.1136/oemed-2022-108608 (PMC10423530; doi:10.1136/oemed-2022-108608)
Supplement: Supplementary data [file oemed-2022-108608supp003.pdf]

BMJ Publishing Group Limited (BMJ) disclaims all liability and responsibility arising from any reliance placed on this supplemental material which has been supplied by the author(s)

Criteria used to grade the quality of evidence in the GRADE evidence tables

Rating of Evidence	Definition
**High ⊕⊕⊕⊕**	Very confident that the true effect lies close to that of the estimate of the effect
**Moderate ⊕⊕⊕◯**	Moderately confident in the effect estimate: the true effect is likely to be close to the estimate of the effect, but there is a possibility that it is substantially different.
**Low ⊕⊕◯◯**	Confidence in the effect estimate is limited: the true effect may be substantially different from the estimate of the effect.
**Very Low ⊕◯◯◯**	Very little confidence in the effect estimate: the true effect is likely to be substantially different from the estimate of effect.

Table 1: Screening and Advice/Referral vs. Screening and CAU/WLC/No Intervention

Certainty assessment							№ of patients		Effect		Certainty	Importance
№ of studies	Study design	Risk of bias	Inconsistency	Indirectness	Imprecision	Other considerations	Intervention	Control	Relative (95% CI)	Absolute (95% CI)		
**Mental Health Symptoms & Disorders**												
**3**	Randomised Trials	Serious[1]	Not serious	Not serious	Serious[2]	None	205	238	Averaging across mental health outcome measures and taking the longest follow-up point in each trial, Pooled SMD = -0.07 [-0.29 to 0.15].		⊕⊕◯◯ **LOW**	CRITICAL

1 This has been rated as serious as one of three trials had high risk of bias, two had some concerns of bias.

2 This has been rated as serious as there were only three small trials, one trial assessing psychological distress, others collapsing between outcome measures (i.e., depression, anxiety). Wide CIs around pooled effect.

Certainty assessment							№ of patients		Effect		Certainty	Importance
№ of studies	Study design	Risk of bias	Inconsistency	Indirectness	Imprecision	Other considerations	Intervention	Control	Relative (95% CI)	Absolute (95% CI)		
**User Satisfaction**												
**2**	Randomised Trials	Serious[3]	Not serious	Very Serious[4]	Serious[5]	None	278[6]	77	Ketelaar et al. (2013) report compliance with interventions referred after screening was 41%, while 13% of participants reported wanting feedback differently. 79% would or would maybe appreciate to be periodically offered the screening intervention in the future. Another trial reported that 76% found the screening intervention informative, 65% reported that it was very or somewhat useful, and 47% agreed that the system reduced their visit time with their doctor (Farzanfar et al., 2011).		⊕◯◯◯ **VERY LOW**	CRITICAL

3 This has been rated as serious as the risk of bias results showed one trial had some-concerns and the other with high risk of bias.
4 This has been rated as very serious as one trial used a study specific survey, not a validated user satisfaction measure. Also limited number of participants who gave user satisfaction data.
5 This has been rated as serious as results not able to be quantitatively assessed. Only proportions from limited samples provided.
6 One trial only gathered user satisfaction data from intervention group only, thus the imbalance in N between intervention and control.

Certainty assessment							№ of patients		Effect		Certainty	Importance
№ of studies	Study design	Risk of bias	Inconsistency	Indirectness	Imprecision	Other considerations	Intervention	Control	Relative (95% CI)	Absolute (95% CI)		
**Work-related Outcomes – Sickness Absence**												
**3**[7]	Randomised Trials	Serious[8]	Serious[9]	Not serious	Serious[10]	None	435	814	At 5-mo follow up, one trial reports the odds of sickness absence in the intervention group being 1.40 times greater than the control group. Another trial found no differences between groups on sickness absence duration at 12-month follow up. Combining these trials resulted in a Pooled SMD = 0.06 [-0.22 to 0.34]. One trial found borderline statistically significantly difference in mean days of sickness absence at long-term follow up (5-years) with a trend favouring the intervention.		⨁◯◯◯ **VERY LOW**	CRITICAL

7 3 different articles, 2 reporting data from the same trial (short (12-mo) and long term (2-5 year) follow-up).
8 This has been rated as serious as trials scored high on risk of bias assessment.
9 This has been rated as serious as one trial found no effect and another found a negative effect of intervention on sickness absence, and I^2= 75.88, indicating substantial heterogeneity.
10 This has been rated as serious due to wide confidence intervals that include the null hypothesis.

Certainty assessment							№ of patients		Effect		Certainty	Importance
№ of studies	Study design	Risk of bias	Inconsistency	Indirectness	Imprecision	Other considerations	Intervention	Control	Relative (95% CI)	Absolute (95% CI)		
**Work-related Outcomes**												
4	Randomised Trials	Serious[11]	Very Serious[12]	Serious[13]	Serious[14]	None	624	995	Pooled analysis of impaired work functioning (2 trials) found a significant decrease favouring intervention (Pooled SMD = -0.26 [-0.48 to -0.04]) at 3-month follow up, which was maintained at 6-month follow up (Pooled SMD = -0.27 [-0.49 to -0.05]). At 5-month follow-up another trial found productivity to be significantly better in the control condition (SMD = -0.19 [-0.36 to -0.02], while at 12-month follow-up another trial found no difference between groups on job satisfaction (SMD = 0.22 [-0.19 to 0.63]). Pooling these, the effect on positive work outcomes was -0.03 [-0.42 to 0.36].		⨁◯◯◯ **VERY LOW**	CRITICAL

11 This has been rated as serious as two out of four trials had high risk of bias.

12 This has been rated as very serious as one trial showed a positive effect, two trials showed no effect, one trial showed a negative effect.

13 This has been rated as serious as all data were self-reporting of different outcomes.

14 This has been rated as serious due to wide confidence intervals and effect sizes range from small to moderate effect.

Certainty assessment							№ of patients		Effect		Certainty	Importance
№ of studies	Study design	Risk of bias	Inconsistency	Indirectness	Imprecision	Other considerations	Intervention	Control	Relative (95% CI)	Absolute (95% CI)		
**Adverse Effects**												
-	Randomised Trials	-	-	-	-	-	-	-	-		-	IMPORTANT
**Positive Mental Health**												
2	Randomised Trials	Serious[15]	Not serious	Not serious	Serious[16]	None	120	122	Taking the longest follow-up point in each trial, Pooled SMD = 0.06 [-0.20 to 0.31]		⊕⊕◯◯ LOW	IMPORTANT
**Quality of Life & Functioning**												
**1**	Randomised Trials	Serious[17]	Not serious	Not serious	Very Serious[18]	None	303	683	No effect of intervention reported, but data not presented.		⊕◯◯◯ VERY LOW	IMPORTANT
**Help-seeking**												

[15] 1 trial had overall high risk of bias and another had overall some concerns of bias.
[16] Wide CIs reported in all studies. Only two small trials.
[17] Trial rated with high risk of bias on RoB assessment.
[18] Data not reported thus width of CI or other indices of imprecision cannot be determined.

Certainty assessment							№ of patients		Effect		Certainty	Importance
№ of studies	Study design	Risk of bias	Inconsistency	Indirectness	Imprecision	Other considerations	Intervention	Control	Relative (95% CI)	Absolute (95% CI)		
1	Randomised Trials	Not serious	Not serious	Serious[19]	Serious[20]	None	191	188	One trial found a found a statistically significant effect of study-group time interaction on help-seeking behaviour (SMD = 0.32 [0.02 to 0.62]) at 3-month follow up. However this was attenuated at 6-months (SMD = -0.18 [-0.49 to 0.13]).		⨁⨁◯◯ **LOW**	IMPORTANT

19 This has been rated as serious due to self-report assessment of visiting at least 1 of 11 caregivers (ranging from formal sources i.e., psychologists, to a supervisor or coach).
20 This has been rated as serious due to wide confidence interval calculated using raw data.

Table 2: Screening and Treatment/Intervention vs. Screening and CAU/WLC/No Intervention

Certainty assessment							№ of patients		Effect		Certainty	Importance
№ of studies	Study design	Risk of bias	Inconsistency	Indirectness	Imprecision	Other considerations	Intervention	Control	Relative (95% CI)	Absolute (95% CI)		
**Mental Health Symptoms & Disorders**												
**4**	Randomised Trials	Serious[1]	Serious[2]	Not serious	Not serious	None	592	605	Averaging across mental health outcome measures and taking the longest follow-up point in each trial, Pooled SMD = -0.22 [-0.42 to -0.02]		⊕⊕◯◯ **LOW**	CRITICAL

[1] One of four trials had high risk of bias, two had some low risk of bias.
[2] Rated as serious as moderate heterogeneity was observed (I^2 = 57.59%)

Certainty assessment							№ of patients		Effect		Certainty	Importance
№ of studies	Study design	Risk of bias	Inconsistency	Indirectness	Imprecision	Other considerations	Intervention	Control	Relative (95% CI)	Absolute (95% CI)		
**User Satisfaction**												
**1**	Randomised Trials	Not serious	Not serious	Very Serious[3]	Serious[4]	None	178[5]	0	Ketelaar et al. (2013) report 5% of participants from Boiler et al. (2014) started the e-health interventions. 17% (14 from 82) of participants reported wanting feedback differently. 0% (0 from 4) participants felt following e-mental health intervention helped improve their mental health/work functioning. 33% (23 from 69) would appreciate to be periodically offered the screening intervention in the future.		⨁◯◯◯ **VERY LOW**	CRITICAL

[3] Trial used a study specific survey that was not thoroughly explained, not a validated user satisfaction measure. Also limited number of participants who gave user satisfaction data.
[4] Results not able to be quantitatively assessed between groups. Only proportions from limited samples provided.
[5] Trial gathered user satisfaction data from intervention group only, thus the imbalance in N between intervention and control.

Certainty assessment							№ of patients		Effect		Certainty	Importance
№ of studies	Study design	Risk of bias	Inconsistency	Indirectness	Imprecision	Other considerations	Intervention	Control	Relative (95% CI)	Absolute (95% CI)		
**Work-related Outcomes – Sickness Absence**												
**2**[6]	Randomised Trials	Serious[7]	Not serious	Not serious	Very Serious[8]	None	69	70	Significant intervention effect on sickness absence duration at 12-months (SMD = -0.38 [-0.71 to -0.04]) not maintained at 5-year follow-up (SMD = 0.11 [-0.34 to 0.55]).		⨁◯◯◯ **VERY LOW**	CRITICAL

[6] 2 different articles but 1 trial, each paper reporting data from the same trial (short (12-mo) and long term (2-5 year) follow-up).
[7] Trial scored high on risk of bias assessment.
[8] This has been rated as very serious as there was only one small trial with small sample size (N<200), and wide confidence intervals observed.

Certainty assessment							№ of patients		Effect		Certainty	Importance
**№ of studies**	**Study design**	**Risk of bias**	**Inconsistency**	**Indirectness**	**Imprecision**	**Other considerations**	**Intervention**	**Control**	**Relative (95% CI)**	**Absolute (95% CI)**		
**Work-related Outcomes**												
3	Randomised Trials	Serious[9]	Very Serious[10]	Serious[11]	Serious[12]	None	523	535	Pooled intervention on positively valanced work-related outcomes (combined measures including productivity, job satisfaction, work ability, and engagement) taking longest follow-up from each study was SMD = 0.24 [-0.04 to 0.52].		⨁◯◯◯ **VERY LOW**	CRITICAL

[9] One from three trials had high risk of bias, the other two had low risk of bias.
[10] One trial found no effect and two found positive effects and I^2= 75.88, indicating substantial heterogeneity.
[11] All self-report data assessing different outcomes.
[12] Wide confidence intervals, effect sizes range from null to moderate effect.

Certainty assessment							№ of patients		Effect		Certainty	Importance
№ of studies	Study design	Risk of bias	Inconsistency	Indirectness	Imprecision	Other considerations	Intervention	Control	Relative (95% CI)	Absolute (95% CI)		
**Adverse Effects**												
-	Randomised Trials	-	-	-	-	-	-	-	-		-	IMPORTANT
**Positive Mental Health**												
2	Randomised Trials	Serious[13]	Not serious	Not serious	Serious[14]	None	219	235	Taking the longest follow-up point in each trial, Pooled SMD = 0.14 [-0.04 to 0.33]		⊕⊕◯◯ **LOW**	IMPORTANT
**Quality of Life & Functioning**												
**1**	Randomised Trials	Serious[15]	Not serious	Not serious	Very Serious[16]	None	303	683	No effect of intervention reported, but data not presented.		⊕◯◯◯ **VERY LOW**	IMPORTANT
**Help-seeking**												
**-**	Randomised Trials	-	-	-	-	-	-	-	-		-	IMPORTANT

[13] One trial had overall high risk of bias and another had low risk of bias.
[14] Wide confidence intervals observed crossing the null.
[15] Trial rated with high risk of bias on RoB assessment.
[16] Data not reported thus width of CI or other indices of imprecision cannot be determined.
